# Predicting Outcomes of Preterm Neonates Post Intraventricular Hemorrhage

**DOI:** 10.3390/ijms251910304

**Published:** 2024-09-25

**Authors:** Gabriel A. Vignolle, Priska Bauerstätter, Silvia Schönthaler, Christa Nöhammer, Monika Olischar, Angelika Berger, Gregor Kasprian, Georg Langs, Klemens Vierlinger, Katharina Goeral

**Affiliations:** 1 Center for Health & Bioresources, Competence Unit Molecular Diagnostics, AIT Austrian Institute of Technology GmbH, 1210 Vienna, Austria; gabriel.vignolle@ait.ac.at (G.A.V.); priska.bauerstaetter@ait.ac.at (P.B.); silvia.schoenthaler@ait.ac.at (S.S.); christa.noehammer@ait.ac.at (C.N.); klemens.vierlinger@ait.ac.at (K.V.); 2 Comprehensive Center for Pediatrics, Department of Pediatrics and Adolescent Medicine, Division of Neonatology, Intensive Care and Neuropediatrics, Medical University of Vienna, 1090 Vienna, Austria; monika.resch@kinderarztpraxis-schumanngasse.at (M.O.); angelika.berger@meduniwien.ac.at (A.B.); 3 Department of Biomedical Imaging and Image-Guided Therapy, Division of Neuro- and Musculosceletal Radiology, Medical University of Vienna, 1090 Vienna, Austria; gregor.kasprian@meduniwien.ac.at; 4 Computational Imaging Research Lab, Department of Biomedical Imaging and Image-Guided Therapy, Medical University of Vienna, 1090 Vienna, Austria; georg.langs@meduniwien.ac.at; 5 Computer Science and Artificial Intelligence Lab, Massachusetts Institute of Technology, Cambridge, MA 02139, USA

**Keywords:** biomarker, intensive care, intraventricular hemorrhage, machine learning, neonate, posthemorrhagic hydrocephalus, prediction, prematurity, proteomics, survival

## Abstract

Intraventricular hemorrhage (IVH) in preterm neonates presents a high risk for developing posthemorrhagic ventricular dilatation (PHVD), a severe complication that can impact survival and long-term outcomes. Early detection of PHVD before clinical onset is crucial for optimizing therapeutic interventions and providing accurate parental counseling. This study explores the potential of explainable machine learning models based on targeted liquid biopsy proteomics data to predict outcomes in preterm neonates with IVH. In recent years, research has focused on leveraging advanced proteomic technologies and machine learning to improve prediction of neonatal complications, particularly in relation to neurological outcomes. Machine learning (ML) approaches, combined with proteomics, offer a powerful tool to identify biomarkers and predict patient-specific risks. However, challenges remain in integrating large-scale, multiomic datasets and translating these findings into actionable clinical tools. Identifying reliable, disease-specific biomarkers and developing explainable ML models that clinicians can trust and understand are key barriers to widespread clinical adoption. In this prospective longitudinal cohort study, we analyzed 1109 liquid biopsy samples from 99 preterm neonates with IVH, collected at up to six timepoints over 13 years. Various explainable ML techniques—including statistical, regularization, deep learning, decision trees, and Bayesian methods—were employed to predict PHVD development and survival and to discover disease-specific protein biomarkers. Targeted proteomic analyses were conducted using serum and urine samples through a proximity extension assay capable of detecting low-concentration proteins in complex biofluids. The study identified 41 significant independent protein markers in the 1600 calculated ML models that surpassed our rigorous threshold (AUC-ROC of ≥0.7, sensitivity ≥ 0.6, and selectivity ≥ 0.6), alongside gestational age at birth, as predictive of PHVD development and survival. Both known biomarkers, such as neurofilament light chain (NEFL), and novel biomarkers were revealed. These findings underscore the potential of targeted proteomics combined with ML to enhance clinical decision-making and parental counseling, though further validation is required before clinical implementation.

## 1. Introduction

Premature neonates diagnosed with intraventricular hemorrhage (IVH) are at significant risk of developing posthemorrhagic ventricular dilatation (PHVD), with an estimated incidence rate of about 25% [1]. In this study, we employ cutting-edge targeted proteomic techniques to analyze various liquid biopsy matrices, specifically serum and urine, from a large cohort of 99 neonatal patients prospectively recruited over a 13-year period.

The etiology of IVH is complex, with gestational age (GA) being an independent predictor of IVH risk [1]. In the past decade, much research has focused on the neurodevelopmental outcome of IVH patients, both with and without the development of PHVD. Despite this extensive research [2,3,4], PHVD treatment remains challenging due to missing molecular markers for early detection and the need to strike a delicate balance between the harmful effects of PHVD on the immature brain and the potential risks associated with interventions [1]. Interventions leading to cerebrospinal fluid drainage carry risks of infection and other complications, all of which necessitate careful consideration [5]. Our unit frequently uses extraventricular drainage (EVD) and Ommaya reservoir placement, followed by ventriculoperitoneal shunt.

Notably, PHVD exhibits a strong correlation with neurodevelopmental impairment [6]. Identifying molecular biomarkers that can predict the development of PHVD before clinical symptoms appear could pave the way for potential early interventions.

Biomarkers provide objective measurements in tissue or body fluids and thereby help to predict diseases and disease outcomes, facilitating prevention and treatment in public health and clinical settings [7,8,9,10]. Protein levels measured and detected in minimally invasive liquid biopsies such as serum and urine can be and are used as biomarkers. Mass spectrometry methods struggle with complex biomatrices and the detection of abundant proteins [11,12,13]. Therefore, we opted for a targeted protein detection method, the Proximity Extension Assay (PEA), which combines the specificity of dual antibody recognition with the sensitivity of qPCR (quantitative polymerase chain reaction) readout [14,15]. As previously demonstrated, this technology permits the detection of low-abundance proteins in complex biomatrices [16,17,18,19].

Machine learning (ML) has been used for years in biomedical research to elucidate pathophysiological processes in diseases and identify novel, measurable biomarkers [20]. In the rapidly evolving field of ML, a plethora of modeling methods and functions have emerged from diverse domains, including statistics [21], regularization ML [22], deep learning [23], decision trees [24], and Bayesian [25] approaches. The sheer abundance of techniques poses a challenge in identifying the single best method for a specific task. A solution is to combine the strengths of various approaches into ensembles, leveraging the advantages of multiple methodologies [20]. Recent studies have already demonstrated the efficacy of ensemble methods, particularly in the context of feature selection [26,27,28]. These ensembles have shown remarkable performance in terms of both stability and prediction accuracy [29]. The use of different ML methods for feature selection offers several advantages. Firstly, it allows for the exploitation of complementary information provided by different feature selection methods, capturing diverse aspects of the underlying data distribution. Secondly, they can mitigate the risk of overfitting and enhance generalization, as they provide a collective decision-making process that weighs the consensus of multiple models. Thirdly, the inclusion of multiple methods contributes to increased robustness against noise and uncertainties present in real-world datasets. We chose to synthesize the feature selection methods from various ML models for our final biomarker selection, thereby leveraging the benefits of ensemble feature selection.

The primary objective of our study was to provide novel insights, identify clinically relevant biomarkers, and explore patient group differences within clinically defined time frames. We hypothesized that prediction of PHVD and survival is possible based on targeted proteomic data, resulting in ML models from which we can extract novel biomarkers. The main contributions of our study are a rigorous ML setup for analyzing PEA data for biomarker discovery and a set of 41 predictive proteins that will fuel future molecular research in the field of pediatrics and hold potential practical implications in early PHVD detection and prevention.

## 2. Results

### 2.1. Cohort and Sample Description

Our prospectively enrolled cohort consisted of 99 patients with IVH, from whom we collected a total of 1109 liquid biopsies (591 serum and 518 urine samples; Table 1, Table 2, Tables S1 and S2). The overall survival rate of the cohort was 70.7% (Table 2). The highest rate of neonatal mortality occurred within the first month of life (75.9% of patients died within one month).

### 2.2. Exploratory Data Analysis

Out of 111,592 individual Olink measurements, only four led to missing values. We analyzed a heatmap based on the uncorrected NPX values and visualized the data through score plots of the first two principal components of a PCA (Figures S1, S2, S5 and S6). After correcting for the batch effect of the plates, we re-examined the visualized data in a heatmap (Figures S3 and S7). Following the removal of positive and negative controls, we performed a PCA on the batch-corrected NPX values (Figures S4 and S8). We did not detect a distinct pattern or clustering in the score plots based on visual data inspection. When comparing the PCA in serum samples with the colored events PHVD, PHVDp1, PHVDp2, and PHVDp3, we detected a tendency in the second component (from top towards bottom) (Figure S9).

Following batch correction, a comparison between patient groups at each event was performed. Upon examining the adjusted *p* values for all possible comparisons between PHVD positive (PHVDp) and PHVD negative (PHVDn) patients in the urine dataset, we identified only one significant (threshold adjusted *p* value < 0.05) adjusted *p* value of 0.018 for the ADAM15 (Disintegrin and metalloproteinase domain-containing protein 15) protein at 32 weeks, as detailed in Table S3. Conversely, when comparing the different timepoints, we observed highly significant changes in protein expression levels (Table S4). The PAEP (Glycodelin) protein showed significantly different levels, with an adjusted P value, in 38.1% of all timepoint comparisons in the urine data (Table S4). When analyzing the neurofilament light chain (NEFL) levels at the IVHp timepoint (Table 1) in serum samples, we observed a significant log2-fold change (*p* value = 0.0237) of 0.61 between PHVDp and PHVDn patients (Table S5). Moreover, in the comparison of different timepoints within the serum data, PSG1 (Pregnancy Specific Beta-1-Glycoprotein 1) emerged as the most significant protein. It showed an adjusted P value of 0.0002 in the comparison between the IVH and IVHp events, with a log2-fold change of 2. The differentially regulated proteins in serum and urine vary over time and between patient groups, indicating distinct biological processes and highlighting the dynamic nature of protein expression.

Upon reviewing the adjusted P values for all possible comparisons between surviving and deceased patients, we identified several proteins in both matrices (Tables S7 and S8). In serum samples, the cytokine interleukin-15 (IL-15) was the most significantly detected protein at the IVHp timepoint (adjusted *p* value = 0.0003, log2-fold change = −0.88). At the PHVD timepoint, 33 significant proteins were identified. Notably, NEFL exhibited a highly significant log2-fold change (adjusted *p* value = 8.82 × 10^−8^) of −1.89. This indicates a roughly 13-fold higher concentration in deceased patients compared to those from surviving patients. At the same timepoint, ADAM15 was found in significantly higher quantities in surviving patients, with levels 3.96 times higher than in deceased patients (Table S8).

### 2.3. Machine Learning Reveals Potential Novel Biomarkers

We evaluated 500 models trained for PHVD prediction and 1100 models trained to predict survival outcomes. We decided to evaluate only those models that met our predefined thresholds and then selected features from these models that displayed a variable importance ≥ 50 on a 0–100 scale. We performed this selection process for all trained models across all defined events (Table 3). All models were trained to optimize their hyperparameters to maximize AUC while keeping sensitivity at a minimum of 0.6. By following this method, we ensured that the models maintained a robust balance between predictive performance and practical utility in a clinical setting. This approach mitigated the risk of overfitting while ensuring that models maintained an acceptable level of true positive detection (sensitivity ≥ 0.6), which is crucial in scenarios where false negatives carry significant consequences. Additionally, optimizing for area under the curve (AUC) facilitated the evaluation of overall model discriminative ability, accounting for both sensitivity and specificity across various decision thresholds. This dual focus on AUC maximization and sensitivity constraint provides a rigorous framework for assessing model efficacy.

**Figure 1:**
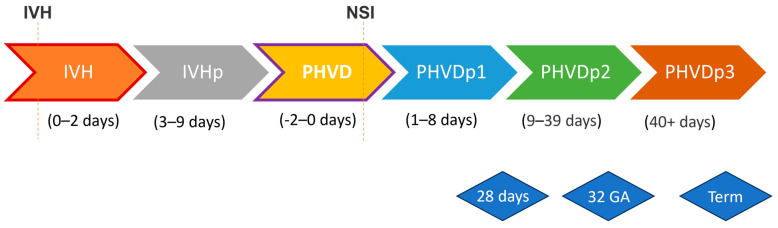
Graphical representation of defined events in a timeline. Dotted red lines intersecting the timeline mark instances of IVH and the corresponding NSI of EVD/Ommaya reservoir. The IVH event encompasses samples collected from the onset of bleeding up to 2 days afterward. The IVHp event spans samples obtained from 3 to 9 days post-bleeding. The PHVD event is delineated by NSI, covering samples from 2 days before the intervention until the intervention itself. Subsequent PHVDp1, PHVDp2, and PHVDp3 events include samples from 1 day after NSI up to 8 days after, 9 to 39 days after, and 40 days or more after NSI, respectively. Comparable timeframes were established for IVH patients without the development of PHVD/without NSI. The equivalent PHVD event comprises samples from 10 to 18 days after IVH, while the equivalent PHVDp1, PHVDp2, and PHVDp3 events include samples from 10 to 18 days, 19 to 49 days, and 50 days or more after IVH, respectively. Further, we included standard time frames, indicated by blue diamond-shaped rectangles, including 28 days of life (±7 days), 32 weeks after conception (±7 days), and term-equivalent age (GA at sampling time 36.0-41.14).

GA at birth appeared to have a significant contribution in all instances across all models (Table 3, Figures S10 and S11). Conversely, the degree of interventricular bleeding only appeared as a significant variable in the “blood PHVD” model (Table 3), where it did not have the highest variable importance among the selected features. Figure S10 displays the AUC-ROC (A), the prediction distribution (B), and the variable importance (C) for the best-performing algorithm for PHVD prediction. In the analysis of variables selected by the models trained with urine samples from events before PHVD onset (Figure 1), in addition to the reoccurring variable GA at birth, RBKS (Ribokinase) and PPP3R1 (Protein Phosphatase 3 Regulatory Subunit B, Alpha/Calcineurin Subunit B Type 1) were identified as important variables. The models trained with samples collected at the PHVD and IVH_IVHp_PHVD events deliberately included samples from instances where PHVD had already occurred. Again, GA at birth emerged as a prominent variable, alongside other molecular variables like ISLR2 (Immunoglobulin Superfamily Containing Leucine Rich Repeat 2; Table 3). We continued with evaluating the models trained on the serum data to predict the risk of PHVD (Table 3 and Figure S10). The recurring variables selected by the models for the IVH, IVHp, and IVH_IVHp events included GA at birth, PPP3R1, and FUT8 (Alpha-(1,6)-fucosyltransferase). Additionally, RBKS was also selected by models trained with samples from the IVHp event. The serum models, trained with the samples from the PHVD event, identified ISLR2, as highly important for the prediction of PHVD. Models trained on data from timepoints closer to the PHVD event demonstrated superior performance, as expected.

Evaluation of the models trained to predict patient survival (Figure S11) reveals a distinct pattern, albeit with some similarities to PHVD prediction feature selection. Noteworthy, GA at birth was consistently selected as a predictive variable in all survival models meeting our criteria, based on serum and urine data alike. The urine models identified only a limited number of proteins with sufficient variable importance (Table 3). In contrast, models based on serum data selected a wider array of features (Table 3), with recurring features including FGFR2 (fibroblast growth factor receptor 2) and IFNL1 (Interferon Lambda 1), among others.

Based on all evaluated ML models, 41 significant uncorrelated protein markers displayed predictive power.

### 2.4. Canonical Correlation Analysis Discloses Unexpected Independence

**Figure 2:**
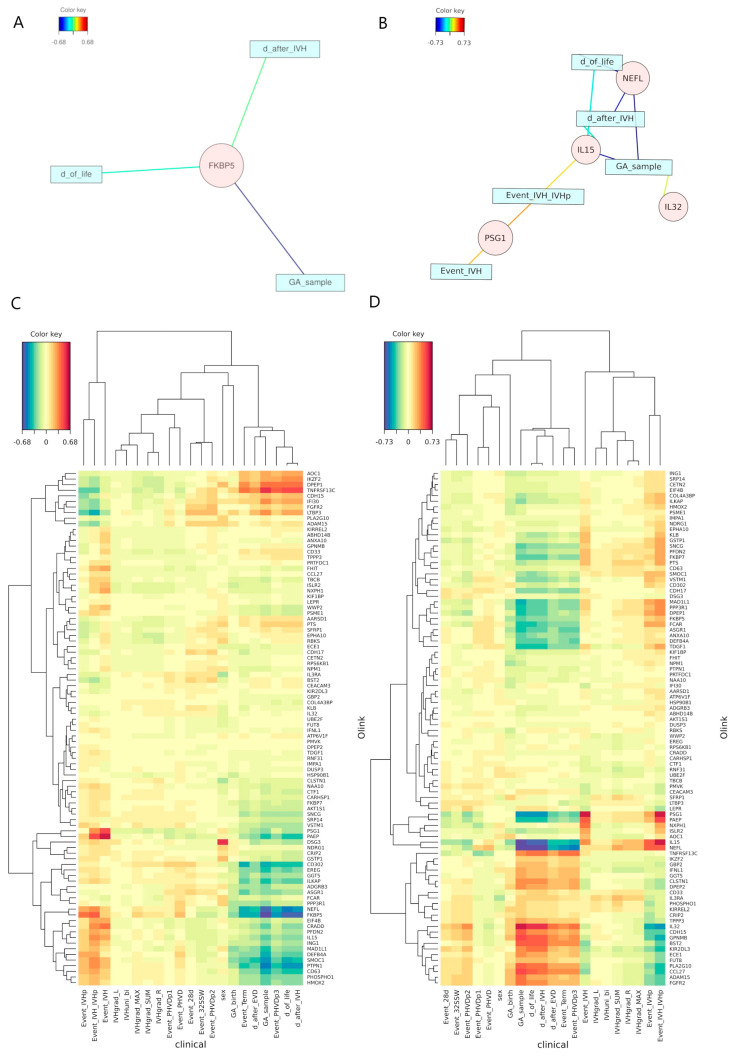
(**A**)-visualization of the relevance associations network based on the rCCA between distinct clinical datasets characterizing the samples and the Olink measurements based on the urine data. The applied correlation threshold for inclusion in both networks was set to 0.6. (**B**)-visualization of the relevance associations network based on the rCCA between distinct clinical data sets characterizing the samples and the Olink measurements based on the serum data. (**C**)-heatmap of the rCCA between distinct clinical datasets characterizing the samples and the Olink measurements based on the urine data. The dendrogram in the heatmap was computed with the complete linkage method to find similar clusters based on the Euclidean distance. (**D**)-heatmap of the rCCA between distinct clinical datasets characterizing the samples and the Olink measurements based on the serum data. The dendrogram in the heatmap was computed with the complete linkage method to find similar clusters based on the Euclidean distance. All clinical data beginning with “Event_” indicate the samples belonging to a specific timeframe; these timeframes are highly correlated to features such as “day of life”, “day after IVH” or “GA of the sample”.

To elucidate and validate the attributes identified by the ML models, we conducted an rCCA on the clinical and molecular data. rCCA was selected due to the high dimensionality of the dataset, where the number of variables exceeds the number of experimental units, making the computation of the covariance matrix inverse intractable without the application of regularization techniques. We displayed the relevance associations network for the rCCA (Figure 2A,B), with the inherent advantage of simultaneously representing both positive (red) and negative (blue) correlations. The applied correlation threshold for inclusion in the network was set to 0.6. Moreover, we visualized the results as a heatmap to give an overall view of the results (Figure 2C,D). As shown, only significant correlations associated with temporal parameters are discernible. A significant negative correlation between NEFL and temporal parameters in serum indicates a decrease over time. Inversely, a positive correlation between samples collected within the IVH timeframe and PSG1 suggests increased levels at earlier timepoints (Figure 2).

Additionally, we examined the correlation between GA at birth and the maximum degree of IVH, calculating the Pearson (r = −0.24), the Spearman correlation coefficient (ρ = −0.24), and the point-biserial correlation (r_pbis_ = −0.25, *p*-value = 0.01). These results suggest no strong, significant correlation between the categorical and continuous variables, indicating their independence based on our collected data.

## 3. Discussion

The role of molecular factors in preventing PHVD in patients with IVH remains largely unexplored, yet identifying these factors could be pivotal in advancing clinical care. By recognizing molecular signatures, we may be able to develop predictive biomarkers that not only improve early diagnosis but also serve as therapeutic targets, potentially mitigating adverse outcomes in premature neonates. IVH is a major complication in preterm infants, significantly elevating mortality risk. However, its direct association with mortality remains uncertain due to the frequent presence of other life-threatening comorbidities that complicate patient outcomes [30]. This underscores the need for a multifaceted approach to clinical management that extends beyond simply addressing the hemorrhage itself. It is important to clarify that the primary focus of our study was not to predict IVH occurrence. Rather, we sought to identify early biomarkers that could predict the progression of IVH to PHVD. We also acknowledge that if intracranial pressure (ICP) is elevated to the point of requiring neurosurgical intervention, predicting PHVD may become redundant, as surgical treatment would already be indicated. Nonetheless, our study fills a critical gap by focusing on the identification of potential biomarkers that could predict PHVD before the onset of raised ICP, ultrasonographic signs, and clinical indications for intervention. Furthermore, we highlight the distinction between our approach and traditional methods used in adult hydrocephalus after shunting that rely on intracranial compliance (ICC) as an index for evaluating hydrocephalus. ICC is commonly assessed through imaging and ICP measurements after hydrocephalus has already developed, as discussed in the referenced study [31]. While ICC provides valuable insights into disease progression after hydrocephalus onset, it does not offer early predictive value for pre-symptomatic intervention. Our research aims to address this gap by identifying molecular markers that can predict PHVD development prior to the manifestation of clinical or ultrasonographic symptoms and raised ICP. In the broader context of clinical practice, the current reliance on imaging limits early intervention opportunities. By leveraging targeted proteomics and machine learning, our study contributes a novel approach to biomarker discovery, potentially transforming the clinical management of IVH. The identification of early predictive biomarkers could not only enhance early diagnosis and treatment decisions but also guide therapeutic development aimed at preventing PHVD and improving survival outcomes in neonates. This positions our research within the evolving landscape of clinical prediction and underscores its potential significance in advancing neonatal care.

The study strengths include the rigorous evaluation of ML models, access to an exceptional cohort of 99 neonates, and the amount of unprecedented molecular information, providing us with invaluable data. Exploratory analysis techniques revealed significant differences in ADAM15 expression between PHVDp and PHVDn patients in urine samples, whereas other comparisons yielded no significant adjusted *p* values. However, comparing different timepoints in urine, we observed highly significant changes in protein expression levels across various events, with PAEP and PSG1 being the most notable. PAEP is mainly expressed in the endometrium and the placenta, while PSG1 is strongly expressed exclusively in the placenta [32]. The detection of these pregnancy-linked proteins may be attributed to the transfer from the mother to the fetus, undergoing secretion, metabolism, and eventual removal from the system of premature infants post-birth. Significant differences were observed when comparing surviving and deceased patients at different timepoints. For example, at the PHVD timepoint, serum NEFL levels in deceased patients were approximately 13 times higher than in survivors, while ADAM15 levels were significantly higher in survivors, suggesting NEFL as a predictor for mortality, while ADAM15 might play a role in the protection thereof. As previously published, the metalloproteinase ADAM15 is upregulated by shear stress, promoting endothelial cell survival via KLF2-induced expression [33]. Knockdown of ADAM15 reduces survival under flow conditions by 6.7-fold, highlighting its protective role. In contrast, the absence of ADAM15 at low shear stress or static conditions leads to increased endothelial damage and vascular inflammation [33]. Additionally, ADAM15 expression is elevated in lung CD8(+) T cells, macrophages, and bronchial epithelial cells in COPD patients, where it inversely correlates with airflow obstruction, indicating its broader protective role in both vascular and pulmonary pathologies [34].

rCCA identified significant correlations between time-related variables and molecular markers. Notably, NEFL strongly correlated with patient age in serum data, and FKBP5 (FK506-binding protein 5-prolyl isomerase) with postnatal age in urine data. FKBP5 has previously been found to be associated with physical and psychological stress [35,36], which might explain the negative correlation to patient age in preterm infants with IVH. Interestingly, no significant correlation was found between GA at birth and the degree of IVH, contrary to the expectation that a more immature brain would be more susceptible to severe bleeding. This underscores the complexity of the multifactorial condition IVH and the need for further research into its underlying mechanisms.

In all evaluated ML models, GA at birth had high or the highest variable importance, contributing significantly to predicting PHVD and survival. Clinically, this reinforces the importance of considering GA at birth alongside other clinical variables in risk stratification and treatment decision-making for IVH patients. It was highly unexpected to find that the degree of IVH did not significantly contribute to the models predicting PHVD. As the variables GA at birth and IVH degree were not strongly correlated, we were able to rule out a potential influence on the variable selection process. Nevertheless, the selection of GA at birth as a predictive variable has a clear rational: it reflects the level of development of immature patients and their capacity to repair damage resulting from IVH [37]. Another plausible explanation could be the deficit in compensating and regulating cerebrospinal fluid pressure in more immature preterm patients. These findings suggest that GA at birth has a stronger impact on patient outcomes than the degree of IVH.

The selection of serum ISLR2 in models post-PHVD development indicates distinct molecular processes differentiating PHVDp and PHVDn patients [38]. ISLR2, expressed in the brain and testis [38], is linked to congenital hydrocephalus [38], and predicted to be involved in the positive regulation of axon extension during neural development. Serum ISLR2 shows decreased NPX values shortly after the PHVD event in PHVDp patients. Models trained on the events preceding PHVD identified serum PPP3R1, FUT8, and RBKS as important variables, suggesting PP3R1′s protective role against PHVD. Also, increased concentrations in urine, indicating higher excretion, could indicate a potential imminent PHVD.

To discern the pathophysiological mechanisms explaining the different concentrations of these predictive markers, more research is needed. We identified predictive biomarkers in both serum and urine that contribute to both the development of PHVD and their protection against it. This discovery paves the way for novel targets for pharmaceutical interventions, enabling more precise monitoring and prediction of patients, particularly those at risk. This advancement could help in deciding whether invasive procedures are indicated in an early stage, a decision that, until now, could not be easily made. Our results indicate the ability to predict PHVD development at an early stage, before detectable ventricular dilatation and the clinical manifestation thereof. This may be due to noticeable molecular microprocesses in the developing brains of neonates, indicating pathophysiological changes before clinical symptoms appear.

Survival prediction models using urine data were limited to 3 due to their failure to meet our inclusion thresholds. This limitation can be attributed to the median day and GA at death in deceased patients. The models included showed one prevalent variable in common, which was GA at birth. Remarkably, looking at the survival models trained on serum, we identified several molecular variables pointing towards complex processes. Additionally, GA at birth, we defined IFNL1 and FGFR2 as protective biomarkers and indicators of survival. As IFNL1 [39,40] plays an important role in the immune system, we assume that the protective features are involved in a more resistant immune response to subclinical infection processes [41]. Given FGFR2′s role in cell mitosis and differentiation, we can infer that its protective abilities might be linked to repair systems activated post-bleeding. We successfully identified the known biomarker NEFL, previously confirmed as a predictor for outcome in IVH patients [42], thereby verifying our approach since NEFL is used as a proxy for neuronal damage [43,44,45].

## 4. Materials and Methods

### 4.1. Sample Collection

We prospectively enrolled neonatal IVH patients over a 13-year period, from May 2011 to March 2023, and collected liquid biopsies, comprising serum and urine. Sample collection and processing followed a uniform protocol throughout the study. Samples were collected in appropriate tubes, immediately cooled, transferred to the central pediatric laboratory within 24 h for centrifugation, distributed into aliquots, and stored at −80 °C for batchwise analysis. Samples were categorized according to clinically defined timeframes and standard time windows (Figure 1).

### 4.2. Targeted Proteomics

Protein expression was measured using PEA technology, specifically employing the Olink^®^ Target 96 Neuro Exploratory Panel, as described previously [14,15]. Normalized Protein eXpression (NPX) values, Olink’s arbitrary unit, in log2 scale and inversely related to the Ct-value, were used for relative quantification only. We measured a total of 92 protein analytes per sample, with each measurement requiring 1 µL of sample. Due to the very low level of missing data, we employed principal component analysis (PCA) to impute missing values.

### 4.3. Machine Learning and Biostatistical Analysis

We conducted an exploratory analysis to determine the suitability of data from different biological matrices for subsequent analysis. In this more descriptive approach to the data, we applied univariate statistical methods, linear and logistic regression, dimensionality reduction, and clustering analyses. Foremost, we estimated the variability of experimental effects, including the sample batch, with a principal variance component analysis (PVCA). The approach leverages the strengths of a PCA to efficiently reduce data dimension while maintaining most of the variability in the data and variance components analysis, which fits a mixed linear model using factors of interest as random effects to estimate and partition the total variability. Detected batch effects were corrected using the ComBat function from the sva R package [46,47]. We detected a batch effect between the plates in both the urine and serum data, with 0.11 and 0.034 weighted average proportion of variance, respectively. After removing the batch effect and conducting another PVCA, we found a reduction to 0.046 (58.2%) and 0.007 (79.4%). To investigate the changes occurring between the defined events, we conducted a group comparison using the limma R package [48] to identify significant changes in the NPX values (see Table 1). We compared samples from patients with PHVD (PHVD positive, PHVDp) to those without (PHVD negative, PHVDn) and survivors to deceased patients at each timepoint. Additionally, we compared all time points against each other to identify any changes. These analyses were based primarily on statistical analysis with various R packages provided by the biocomputing platform Bioconductor [47,48]. Considering the evidence presented in the manuscript and the significant signals measured, we concluded that urine samples are suitable for further biomarker detection.

Next, we addressed the question of whether it is possible to predict patient outcomes based on the NPX measurements and a thorough evaluation of phenotype data. We used our ML models to identify patients with IVH who are at risk of developing PHVD and to predict patient survival. In this set of ML models, we included GA at birth, as this has previously been shown to significantly predict the risk of certain outcomes [49] and the maximum grade of IVH. Notably, they were also trained for each event individually (Table 1). Five different supervised ML classification models were used to address the limitations of relying on one single algorithm for biomarker detection. The supervised form of a partial least squares discriminate analysis (PLS-DA) was applied to each training dataset timepoint independently [21,50,51,52]. The machine-learning algorithm random forests (RF) analysis was applied independently of the PLS-DA analysis to the same dataset [52,53,54,55]. The third algorithm used was an Elastic-Net Regularized Generalized Linear Model (GLMnet), used to fit generalized linear and similar models via penalized maximum likelihood. This fast algorithm further removes degeneracy and wild behavior caused by extreme correlations [56]. We further fitted a neural network model (multilayer feed-forward supervised network) to the datasets [57]. Finally, we applied a Naïve Bayesian (NB) supervised algorithm. To estimate the variable importance, an inbuilt function of the caret function was used for the approximation of the relative measure of the variable importance calculated on the area under the receiver operating curve (AUC-ROC) and the R2 statistic [58]. These five models were used to determine the importance of the 92 proteins included in the panel to discriminate PHVDp from PHVDn patients and to predict their survival. As all these different models utilize different metrics to determine variable importance and therefore cannot be compared directly, we decided to use normalized scaled metrics for each predictor in each fitted model, as included in the caret R package. In this system, a score of 100 represents the highest importance to the model in deriving a classification [58]. Variables with variable importance values > 50 were considered to contribute significantly to the model. In the following analysis, each model type (PLS-DA, GLMnet, RF, Neural Network, and NB) was performed 10 times on 10 randomly split test and training sets, which were submitted to a 10-fold cross validation repeated 10 times each [21,32]. The resulting performance metrics were then summarized for each model algorithm at a given event to ensure a constant result independently from the split of the data in training and test sets. For the final selection of variables from the models, we applied the following thresholds: the model had to achieve an AUC-ROC of ≥0.7, sensitivity ≥ 0.6, and selectivity ≥ 0.6. Moreover, the variables had to score a variable importance mean of 50 or higher. We only included gestational age at birth and the degree of IVH in the models, focusing solely on the identification of molecular markers and assessing their reliability without the influence of too many additional variables. The aim was to identify disease-specific proteins and their strength in discriminating between different patient groups. These models were separately trained on urine and serum data. Visualizations and further statistical analyses were performed in the R environment [59,60].

To verify and explain the feature selection in the ML models, we performed a regularized canonical correlation analysis (rCCA) using the mixOmics R package, aimed to identify potential correlations between two multivariate data matrices: the clinical data describing the samples and the proteomics data [61,62,63]. rCCA was chosen due to the large number of variables compared to the experimental units, and therefore calculating the inverse for these would be impossible without regularization.

## 5. Conclusions

Our study provides the first glimpses into molecular processes driving changes in preterm neonates with IVH, marking a significant contribution to understanding the early stages of this condition. Thus, enhancing our knowledge of the molecular drivers of IVH progression, while practically, these findings hold promise for earlier diagnosis of high-risk individuals. This could lead to preemptive treatments aimed at preventing the development of PHVD. Clinically, the ability to predict PHVD early has the potential to transform patient care, especially in medical centers across the US. Early identification could prompt more frequent ultrasound screenings, allowing for timely interventions, such as lumbar punctures, thereby reducing the need for invasive neurosurgical procedures and improving outcomes. Our findings, particularly the role of GA at birth as the most powerful predictor in machine learning models, underscore the importance of this metric in managing IVH patients. Despite these advances, our study is limited by the relatively small sample size and the need for larger, multicenter studies to validate our predictive models. Future research should explore the integration of additional biomarkers to refine risk stratification and investigate longitudinal outcomes following early interventions. Given the severity of this condition, we are grateful for the participation of all patients and caregivers in this study.

## Figures and Tables

**Table 1:** Overview of clinically defined timeframe events.

Defined Event	Sampling Timeframe	Serum Samples	Urine Samples	Median Day of Life (IQR)
IVH	0 to 2 days after IVH (bleeding Event)	72	52	3 (2–4)
IVHp	3 to 9 days after IVH and <−2 days after NSI	108	101	6 (5–9)
PHVD	−2 to 0 days after NSI for PHVD positives; equivalent timeframe: 10 to 18 days after IVH for PHVD negatives	99	78	14 (11–17)
PHVDp1	1 to 8 days after NSI for PHVD positives; equivalent timeframe: 10 to 18 days after IVH for PHVD negatives	96	109	18 (15–22)
PHVDp2	9 to 39 days after NSI for PHVD positives; equivalent timeframe: 19 to 49 days after IVH for PHVD negatives	108	83	38 (30–46)
PHVDp3	40+ days after NSI for PHVD positives; equivalent timeframe: 50+ days after IVH for PHVD negatives	131	120	79 (67–96)
IVH_IVHp	0 to 9 days after IVH	172	152	5 (3–8)
28 days of life	21 to 35 days of life (28 days ± 7 days)	82	83	27 (24–30)
32 weeks	31.0 to 33.0 GA (32 weeks ± 7 days)	59	54	39 (22–50)
term-equivalent age	predicted birth timepoint/discharge from clinic, 36.0 to 41.14 GA	93	84	83 (70–96)
Defined time windows: a single sample can be classified into two or more events.				

Abbreviations: IQR, inter-quartile range; NSI, neurosurgical intervention; PHVD, posthemorrhagic ventricular dilatation; IVH, interventricular hemorrhage; GA, gestational age.

**Table 2:** Clinical data of the patients included in this study.

	PHVDn (n = 46)	PHVDp (n = 53)	Total (n = 99)	*p* Value
Survival				0.045 ^a^
Deceased n (%)	18 (39.1)	11 (20.8)	29 (29.3)	
Survived n (%)	28 (60.9)	42 (79.2)	70 (70.7)	
Median day at death (IQR) (days)			17 (10–25)	
Median GA at death (IQR) (weeks)			26.57 (25.57–29.14)	
GA at birth				<0.001 ^b^
Median (IQR) (weeks)	24.43 (23.57–25.96)	26.29 (25.29–28.14)	25.57 (24.14–27.14)	
Range	23.00–29.71	23.29–33.29	23.00–33.29	
Sex male n (%)	30 (65.2)	35 (66.0)	65 (65.7)	0.932 ^a^
IVHgrade_L				
Median (IQR)	3 (2–4)	3 (3–3)	3 (2–4)	0.167 ^b^
0 n (%)	5 (10.9)	1 (1.9)	6 (6.1)	0.002 ^a^
2 n (%)	16 (34.8)	8 (15.1)	24 (24.2)	
3 n (%)	11 (23.9)	32 (62.3)	44 (44.4)	
4 n (%)	14 (30.4)	11 (20.8)	25 (25.3)	
IVHgrade_R				
Median (IQR)	3 (2–4)	3 (3–3)	3 (2–4)	0.138 ^b^
0 n (%)	5 (10.9)	1 (1.9)	6 (6.1)	0.042 ^a^
1 n (%)	3 (6.5)	1 (1.9)	4 (4.0)	
2 n (%)	12 (26.1)	8 (15.1)	20 (20.2)	
3 n (%)	14 (30.4)	32 (60.4)	46 (46.5)	
4 n (%)	12 (26.1)	11 (20.8)	23 (23.2)	
IVHuni_bi				0.013 ^a^
unilateral n (%)	9 (19.6)	2 (3.8)	11 (11.1)	
bilateral n (%)	37 (80.4)	51 (96.2)	88 (88.9)	
IVHgrade_MAX				
Median (IQR)	3 (2–4)	3 (3–4)	3 (3–4)	0.686 ^b^
2 n (%)	12 (26.1)	2 (3.8)	14 (14.1)	0.002 ^a^
3 n (%)	13 (28.3)	32 (60.4)	45 (45.4)	
4 n (%)	21 (45.7)	19 (35.8)	40 (40.4)	
IVHgrade_SUM				
Median (IQR)	6 (4–6)	6 (6–6)	6 (5–6)	0.040 ^b^
2 n (%)	6 (13.0)	0 (0.0)	6 (6.1)	0.014 ^a^
3 n (%)	3 (6.5)	2 (3.8)	5 (5.1)	
4 n (%)	8 (17.4)	3 (5.7)	11 (11.1)	
5 n (%)	5 (10.9)	6 (11.3)	11 (11.1)	
6 n (%)	13 (28.3)	30 (56.6)	43 (43.4)	
7 n (%)	6 (13.0)	9 (17.0)	15 (15.2)	
8 n (%)	5 (10.9)	3 (5.7)	8 (8.1)	
Number of NSI				<0.001 ^b^
Median (IQR)	NA	3 (2–5)	1 (0–4)	
Range	NA	0.00–10.00	0.00–10.00	
Asphyxia n (%)	10 (21.7)	13 (24.5)	23 (23.2)	0.743 ^a^
NAISor neonatal CSVT n (%)	0 (0.0)	2 (3.8)	2 (2.0)	0.183 ^a^
Encephalitis or ventriculitis n (%)	0 (0.0)	11 (20.8)	11 (11.1)	0.001 ^a^
PDA n (%) ^c^	6 (16.2)	6 (12.0)	12 (13.8)	0.218 ^a^
NEC n (%) ^c^	5 (10.9)	5 (9.4)	10 (10.1)	0.813 ^a^
BPD n (%) ^d^	16 (55.2)	19 (41.3)	35 (46.7)	0.026 ^a^
ROP n (%) ^d^	7 (24.1)	8 (18.2)	15 (20.6)	0.084 ^a^
PVL n (%) ^d^	2 (7.1)	3 (6.8)	5 (6.9)	0.087 ^a^

^a^ *p* Values were calculated using Pearson’s Chi-squared test. ^b^ *p* Values were calculated with a Kruskal –Wallis rank sum test. ^c^ Only diagnosed in survivors as well as deceased patients in case of survival > 34 weeks GA. ^d^ Only diagnosed in survivors as well as deceased patients in case of survival until term. Abbreviations: BPD, bronchopulmonary dysplasia; CSVT, cerebral sinovenous thrombosis; GA, gestational age; IVH, interventricular hemorrhage; IVHgrade_L, degree of IVH in the left brain hemisphere; IVHgrade_MAX, maximum degree of IVH; IVHgrade_R, degree of IVH in the right brain hemisphere; IVHgrade_SUM, summed degree of IVH; IVHuni_bi, unilateral or bilateral IVH; IQR, interquartile range; NAIS, neonatal arterial ischemic stroke; NEC, necrotizing enterocolitis; NSI, neurosurgical intervention; PDA, persistent ductus arteriosus; PHVD, posthemorrhagic ventricular dilatation; PVL, periventricular leukomalacia; ROP, retinopathy of prematurity.

**Table 3:** Features selected based on ML models trained on targeted proteomics.

Model	Features Selected
**Urine Models predicting PHVD**	
Urine IVH	DEFB4A; GA at birth
Urine IVHp	GA at birth; TDGF1
Urine PHVD	– ^a^
Urine IVH_IVHp	RBKS; GA at birth; PPP3R1
Urine IVH_IVHp_PHVD	RBKS; GA at birth; CD33; SNCG; PP3R1
**Serum Models predicting PHVD**	
Blood IVH	PPP3R1; GA at birth
Blood IVHp	FUT8; GA at birth; RBKS
Blood PHVD	KLB; GA at birth; PAEP; PTS; AOC1; ISLR2; NXPH1; IVHgrade_MAX; VSTMT
Blood IVH_IVHp	GA at birth; PPP3R1; FUT8
Blood IVH_IVHp_PHVD	DPEP2; GA at birth
**Urine Models predicting survival**	
Urine IVH	GA at birth; HSP90B1; KIRREL2
Urine IVHp	– ^a^
Urine PHVD	FGFR2; GA at birth
Urine IVH_IVHp	GA at birth
Urine IVH_IVHp_PHVD	– ^a^
Urine PHVDp1	– ^a^
Urine PHVDp2	– ^a^
Urine PHVDp3	– ^a^
Urine 28 days of life	– ^a^
Urine 32 weeks	– ^a^
Urine term-equivalent age	Not able to perform ML
**Serum Models predicting survival**	
Blood IVH	PRTFDC2; GA at birth; AKT1S1; FKBP5; SNCG; DPEP2
Blood IVHp	FGFR2; GA at birth; IL15; FKBP5; DPEP2; CLSTN1; IFNL1; RBKS
Blood PHVD	GPNMB; DSG3; FGFR2; NEFL; IL15; CDH15; ADAM15; GA at birth; KIR2DL3; PLA2G10
Blood IVH_IVHp	DPEP2; GA at birth; IL15; GSTP1; COL4A3BP; PRTFDC1; SNCG
Blood IVH_IVHp_PHVD	GA at birth; DSG3
Blood PHVDp1	FGFR2; ADAM15; NEFL; PLA2G10; IL15; CDH15; BST2; FCAR; GA at birth
Blood PHVDp2	GA at birth; TNFRSE13C; PAEP
Blood PHVDp3	IFNL1; SNCG; GA at birth; TDGF1; ADGRB3; IL32
Blood 28 days of life	– ^a^
Blood 32 weeks	– ^a^
Blood term-equivalent age	– ^a^
Applied thresholds for the models: AUC-ROC ≥ 0.7; Sensitivity ≥ 0.6 and Selectivity ≥ 0.6.Features selected from models passing thresholds had to display a relative variable importance measure ≥ 50.	

^a^ Indicates, that no model passed the threshold for evaluation. Abbreviations: GA, gestational age; IVH, interventricular hemorrhage; PHVD, posthemorrhagic ventricular dilatation; IVHgrade_MAX, maximum degree of IVH.

## Data Availability

The data presented in this study are included in the tables and Supplementary Material. Additional deidentified participant data will not be made available.

## Supplementary Materials

The following supporting information can be downloaded at: https://www.mdpi.com/article/10.3390/ijms251910304/s1.

## Abbreviations

AUC-ROC	area under the receiver operating curve
BPD	bronchopulmonary dysplasia
CSVT	cerebral sinovenous thrombosis
EVD	extraventricular drainage
GA	gestational age
IVH	intraventricular hemorrhage
IVHgrade_L	degree of IVH in the left-brain hemisphere
IVHgrade_MAX	maximum degree of IVH
IVHgrade_R	degree of IVH in the right-brain hemisphere
IVHgrade_SUM	summed degree of IVH
IVHuni_bi	unilateral or bilateral IVH
ML	machine learning
NAIS	neonatal arterial ischemic stroke
NEC	necrotizing enterocolitis
NEFL	neurofilament light chain
NIH	National Institutes of Health
NPX	Normalized Protein eXpression
NSI	neurosurgical intervention
PCA	principal component analysis
PDA	persistent ductus arteriosus
PEA	Proximity Extension Assay
PHVD	posthemorrhagic ventricular dilatation
PHVDp	posthemorrhagic ventricular dilatation positive
PHVDn	posthemorrhagic ventricular dilatation negative
PLS-DA	partial least square discriminate analysis
PVCA	principal variance component analysis
PVL	periventricular leukomalacia
qPCR	quantitative polymerase chain reaction
RF	random forests
ROP	retinopathy of prematurity
rCCA	regularized canonical correlation analysis
VIP	variable importance projection
