# Supplementary material for: Predicting Outcomes of Preterm Neonates Post Intraventricular Hemorrhage
Source: Int J Mol Sci. 2024 Sep 25;25(19):10304. doi: 10.3390/ijms251910304 (PMC11477204; doi:10.3390/ijms251910304)
Supplement: Supplementary file 1 [file ijms-25-10304-s001.zip › ijms-3190406-supplementary.pdf]

Supplementary Online Content

Predicting Outcomes of Preterm Neonates Post Intraventricular Hemorrhage

Gabriel A. Vignolle [1], Priska Bauerstätter [1], Silvia Schönthaler [1], Christa Nöhammer [1], Monika Olischar [2], Angelika Berger [2], Gregor Kasprian [3], Georg Langs [4,5], Klemens Vierlinger [1] and Katharina Goeral [2,*]

[1] Center for Health & Bioresources, Competence Unit Molecular Diagnostics, AIT Austrian Institute of Technology GmbH, 1210 Vienna, Austria
[2] Comprehensive Center for Pediatrics, Department of Pediatrics and Adolescent Medicine, Division of Neonatology, Intensive Care and Neuropediatrics, Medical University of Vienna, 1090 Vienna, Austria
[3] Department of Biomedical Imaging and Image-Guided Therapy, Division of Neuro- and Musculosceletal Radiology, Medical University of Vienna, 1090 Vienna, Austria
[4] Computational Imaging Research Lab, Department of Biomedical Imaging and Image-Guided Therapy, Medical University of Vienna, 1090 Vienna, Austria
[5] Computer Science and Artificial Intelligence Lab, Massachusetts Institute of Technology, Cambridge, MA 02139, USA
* Correspondence: katharina.goeral@meduniwien.ac.at; Tel.: +43-1-40400-323200

Methods S1
Figure S1. Heatmap based on the uncorrected NPX values from the complete set of urine samples (including positive and negative controls).
Figure S2. Score plot of the first two principal components based on a PCA performed on the complete set of uncorrected NPX-values of the urine samples.
Figure S3. Heatmap based on the batch corrected NPX values from the complete set of urine samples (including positive and negative controls).
Figure S4. Score plot of the first two principal components based on a PCA performed on the complete set of batch corrected NPX-values of the urine samples (excluding positive and negative controls).
Figure S5. Heatmap based on the uncorrected NPX values from the complete set of serum samples (including positive and negative controls).
Figure S6. Score plot of the first two principal components based on a PCA performed on the complete set of uncorrected NPX-values of the serum samples.
Figure S7. Heatmap based on the uncorrected NPX values from the complete set of serum samples (including positive and negative controls).
Figure S8. Score plot of the first two principal components based on a PCA performed on the complete set of uncorrected NPX-values of the serum samples.
Figure S9. Four score plots of the first two principal components based on a PCA performed on the complete set of uncorrected NPX-values of the serum samples.
Figure S10. Overview of all best performing model types for the prediction of PHVD, based on urine and serum data separately.
Figure S11. Overview of all best performing model types for the prediction of survival, based on urine and serum data separately.
Table S1. Overview of urine samples.
Table S2. Overview of serum samples.
Table S3. Top 10 differentially expressed proteins comparing PHVDp vs. PHVDn patients in all possible comparisons based on the urine data.
Table S4. Top 10 differentially expressed proteins between different timepoints based on the urine data.
Table S5. Top 10 differentially expressed proteins comparing PHVDp vs. PHVDn patients in all possible comparisons based on the serum data.
Table S6. Top 10 differentially expressed proteins between different timepoints based on the serum data.
Table S7. Top 10 differentially expressed proteins between surviving and deceased patients at defined timepoints based on the urine data.
Table S8. Top 10 differentially expressed proteins between surviving and deceased patients at defined timepoints based on the serum data.
References S1

Methods S1

Biostatistical analysis

In this comprehensive analysis of the data, we employed a range of univariate statistical techniques, including linear and logistic regression, alongside dimensionality reduction and clustering analyses. A principal variance component analysis (PVCA) was used to estimate the variability of experimental effects, including sample batch effects. This method integrates the dimensionality-reducing capabilities of principal component analysis (PCA) with variance components analysis, which utilizes a mixed linear model to treat factors of interest as random effects, enabling the estimation and partitioning of total variance. By applying PVCA, we effectively retained most of the inherent variability in the data while identifying the impact of batch effects. To correct for these batch effects, we utilized the ComBat function from the sva R package [1, 2]. Specifically, we detected batch effects across different plates for both the urine and serum datasets, with weighted average proportions of variance of 0.11 and 0.034, respectively. After applying the batch effect correction, a subsequent PVCA showed a significant reduction in batch-associated variance to 0.046 (a 58.2% reduction) for urine and 0.007 (a 79.4% reduction) for serum. To explore the biological changes between defined clinical events, we performed group comparisons using the limma package [3] to detect significant alterations in NPX values (see Table 1). Comparisons were made between patients with post-hemorrhagic ventricular dilation (PHVD positive, PHVDp) and those without (PHVD negative, PHVDn), as well as between survivors and deceased patients at each timepoint. Additionally, we performed comparisons across all timepoints to assess temporal changes. These statistical analyses were conducted primarily using R packages available through the Bioconductor platform [4, 5], which provided a robust framework for handling the complex data. Based on the evidence and significant signals observed in our study, we conclude that urine samples are highly suitable for further biomarker discovery efforts.

Machine learning

Five different supervised ML classification models were used to address the limitations of relying on one single algorithm for biomarker detection. The supervised form of a partial least square discriminate analysis (PLS-DA) was applied to each training data set timepoint independently [6–9]. The machine-learning algorithm random forests (RF) analysis was applied independently of the PLS-DA analysis to the same data set [10–13]. The third algorithm used was an Elastic-Net Regularized Generalized Linear Model (GLMnet), used to fit generalized linear and similar models via penalized maximum likelihood. This fast algorithm further removes degeneracies and wild behavior caused by extreme correlations [14]. We further fitted a neural network model (multilayer feed-forward supervised network) to the datasets [15]. Finally, we applied a Naïve Bayesian (NB) supervised algorithm. The hyperparameters for each model were selected based on the described cross-validation techniques (see below) to avoid overfitting and to strike an optimal balance between bias and variance. For PLS-DA, the primary hyperparameter tuned was the number of latent components, which controls the complexity of the model. PLS-DA projects the predictors into a lower-dimensional space where maximum covariance between the predictors and response variable is achieved. Selecting an appropriate number of components is critical, as too few components may lead to underfitting, while too many may introduce noise and overfitting. The optimal number of components was determined via cross-validation, maximizing predictive accuracy while preserving model interpretability. In the case of GLMnet, two key hyperparameters were optimized: the penalty parameter α, which controls the trade-off between L1 (lasso) and L2 (ridge) regularization, and the regularization strength parameter λ. The α parameter allows for flexible model fitting by adjusting the sparsity of the solution, while λ dictates the overall amount of shrinkage applied to the model coefficients. Optimal values of α and λ were selected through grid search and cross-validation, ensuring model robustness and preventing overfitting. For RF, the main hyperparameters tuned included the number of decision trees in the forest (n_estimators) and the maximum depth of each tree (max_depth). The number of trees impacts the model's ensemble learning capability, where more trees typically lead to better performance but with higher computational cost. The maximum depth controls the degree of tree complexity, preventing overfitting by limiting the depth at which trees can grow. Additionally, we optimized the minimum number of samples required to split a node (min_samples_split) and to be at a leaf node (min_samples_leaf) to further regulate the model's tendency to overfit the training data. For the Neural Network, we adjusted several hyperparameters to enhance its performance, including the number of hidden layers and neurons per layer, the learning rate, and the activation function. The architecture of the neural network—specifically the number of layers and neurons—determines its capacity to capture complex patterns in the data. The learning rate dictates the speed at which the network adjusts its weights during training, influencing convergence and the risk of falling into local minima. Hyperparameter tuning was performed using a combination of grid search and random search, with model performance evaluated via cross-validation to avoid overfitting. Finally, for the Naive Bayes classifier, the key hyperparameters include the choice of probability distribution for the predictors (e.g., Gaussian, Bernoulli, or multinomial) and the smoothing parameter (α). The Naive Bayes model assumes conditional independence between features, and the choice of distribution is based on the nature of the data (continuous). The smoothing parameter is critical in preventing zero probabilities for unseen feature combinations in the training set, with the optimal value of α determined through cross-validation to balance model accuracy. By systematically tuning these hyperparameters for each model, we ensured that their performance was maximized while minimizing the risk of overfitting. To estimate the variable importance of the models, an inbuilt function of the *caret* function was used for the approximation of the relative measure of the variable importance calculated on the area under the receiver operating curve (AUC-ROC) and the R^2 statistic [16]. These five models

were used to determine the importance of the 92 proteins included in the Panel to discriminate PHVDp from PHVDn patients and to predict their survival. As all these different models utilize different metrics to determine variable importance and therefore cannot be compared directly, we decided to use normalized scaled metrics for each predictor in each fitted model, as included in the *caret* R package. In this system, a score of 100 represents the highest importance to the model in deriving a classification [16]. Variables with variable importance values > 50 were considered to contribute significantly to the model. In the following analysis, each model type (PLS-DA, GLMnet, RF, Neural Network and NB) was performed 10 times on 10 randomly split test and training sets, which were submitted to a 10-fold cross validation repeated 10 times each [17,18]. The resulting performance metrics were then summarized for each model algorithm at a given event to ensure a constant result independently from the split of the data in training and test sets. For the final selection of variables from the models, we applied following thresholds: the model had to achieve an AUC-ROC of ≥ 0.7, sensitivity ≥ 0.6 and selectivity ≥ 0.6. Moreover, the variables had to score a variable importance mean of 50 or higher. We only included gestational age at birth and the degree of IVH in the models, focusing solely on the identification of molecular markers and assessing their reliability without the influence of too many additional variables. The aim was to identify disease-specific proteins and their strength in discriminating between different patient groups. These models were separately trained on urine and serum data. Visualizations and further statistical analyses were performed in the R environment [4, 5].

Additional Information

The survival rates reported in the supplements represent the percentage of survival based on the number of samples collected from patients, not the actual survival rate of the entire patient cohort. The discrepancy arises from the fact that we naturally collected more samples from surviving patients, given that the highest rate of neonatal mortality occurred within the first month of life (75.9% of patients died within one month), biasing the distribution of the samples.

PPP3R1, known for its biased expression in the brain [19], has recently been linked through ML to biomarkers of Alzheimer’s disease [20]. FUT8 is ubiquitously expressed in human tissues[19] and used for diagnosis and prognosis of epilepsy and refractory epilepsy in children[21]. RBKS shows a broad overall expression with some focus in adrenal glands and small intestine tissue [19].

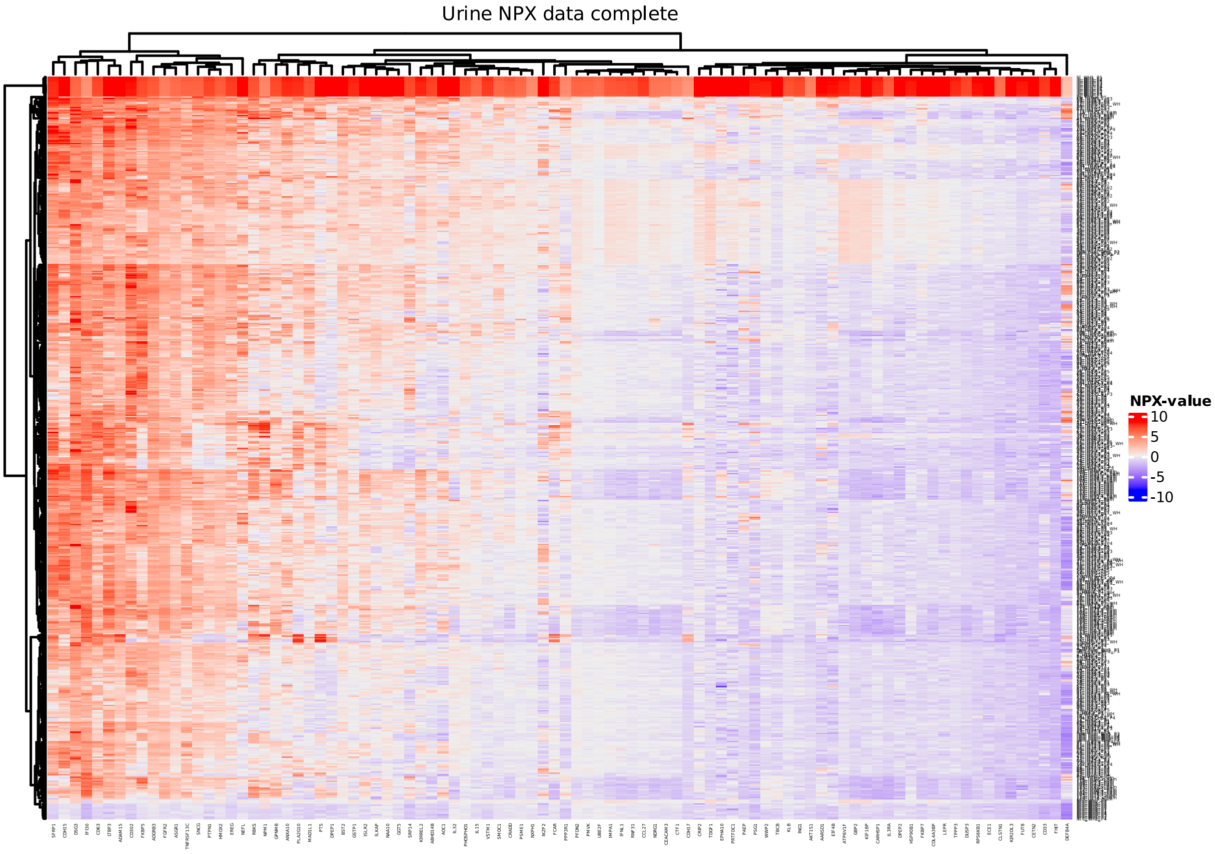

Figure S1: Heatmap based on the uncorrected NPX values from the complete set of urine samples (including positive and negative controls). The dendrogram in the heatmap was computed with the complete linkage method to find similar clusters based on the Euclidean distance.

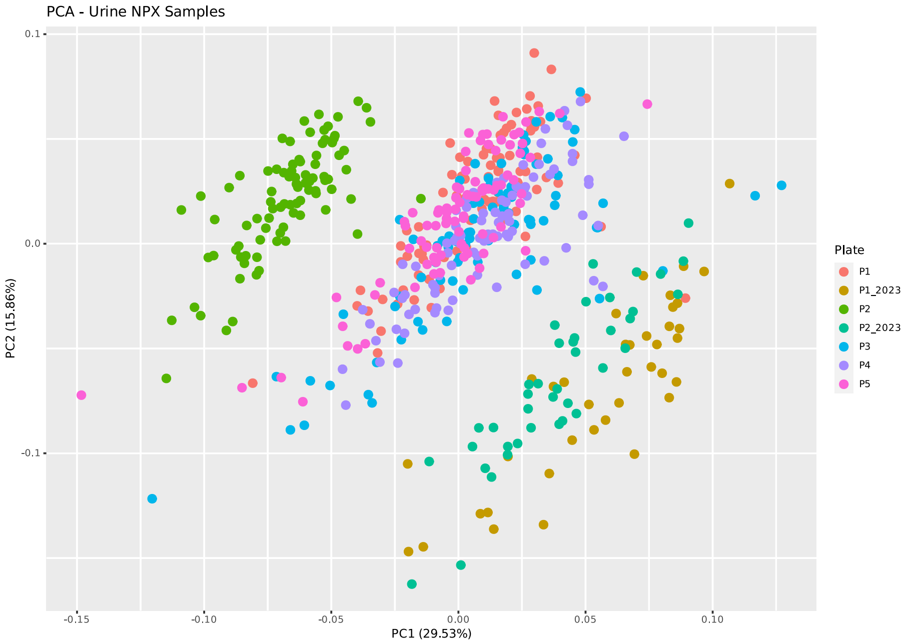

Figure S2: Score plot of the first two principal components based on a PCA performed on the complete set of uncorrected NPX-values of the urine samples. The percentage in the axis labels displays the percentage of the explained variance by this principal component. The dots are colored based on the corresponding Olink plate run.

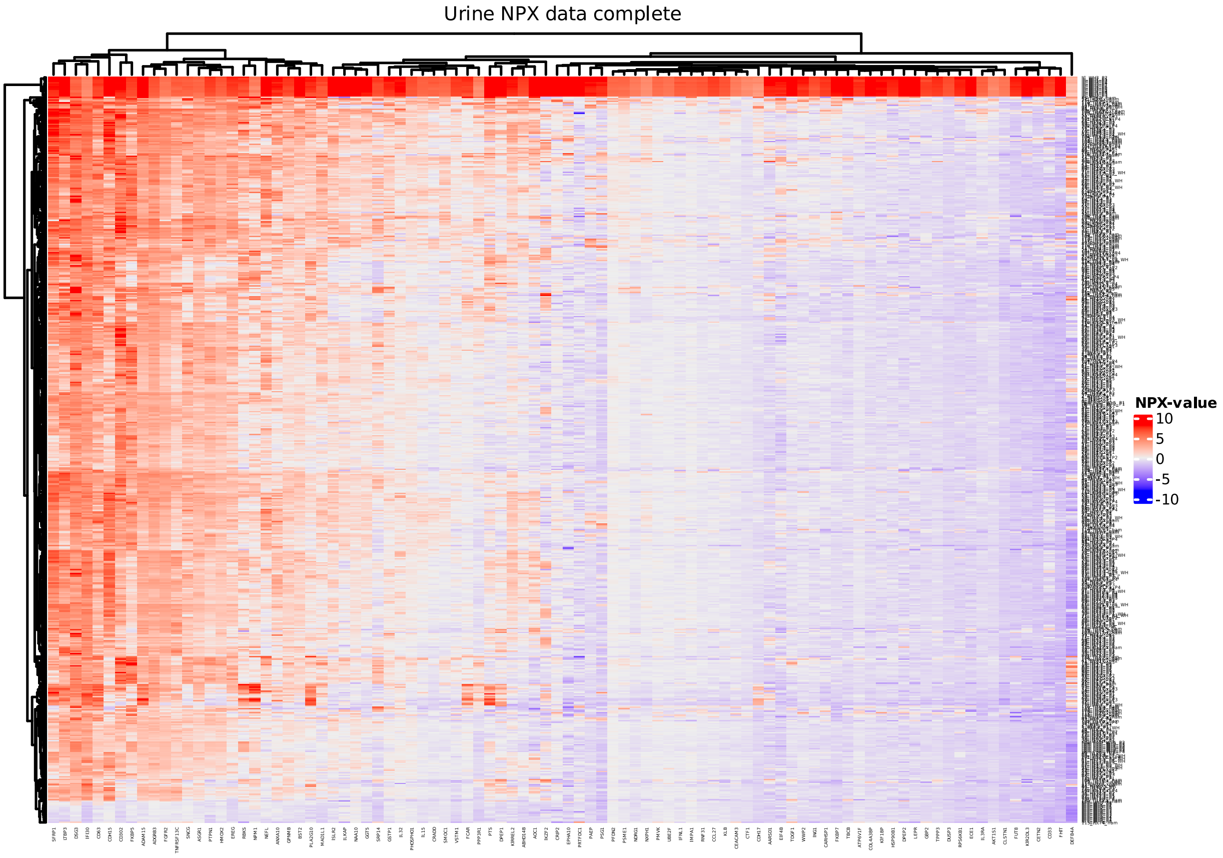

Figure S3: Heatmap based on the batch corrected NPX values from the complete set of urine samples (including positive and negative controls). The dendrogram in the heatmap was computed with the complete linkage method to find similar clusters based on the Euclidean distance.

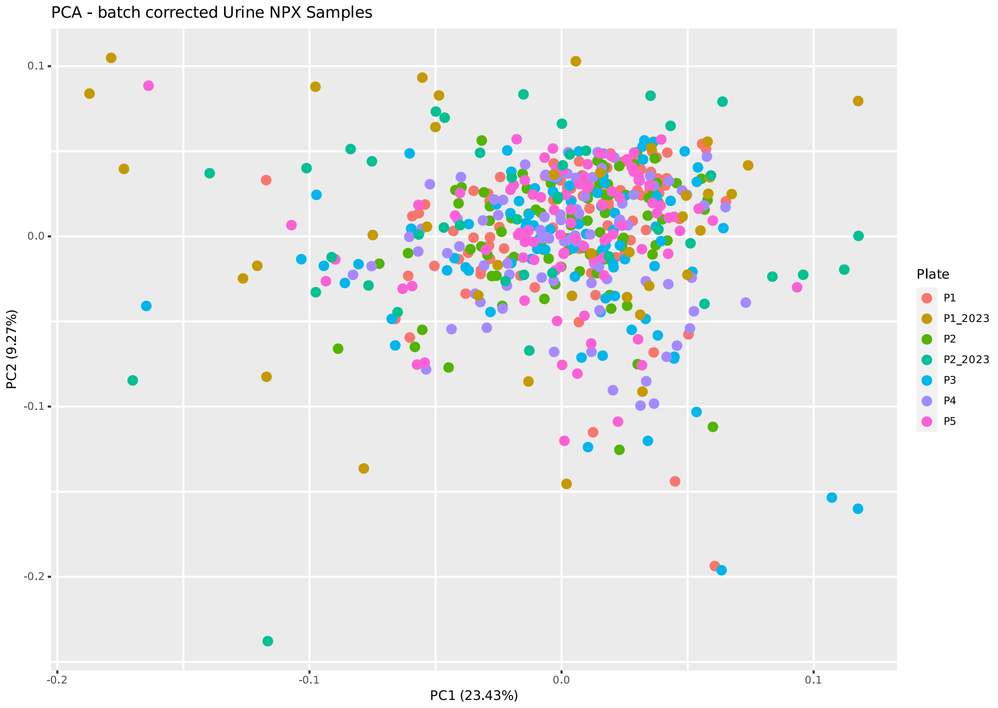

Figure S4: Score plot of the first two principal components based on a PCA performed on the complete set of batch corrected NPX-values of the urine samples (excluding positive and negative controls). The percentage in the axis labels displays the percentage of the explained variance by this principal component. The dots are colored based on the corresponding Olink plate run.

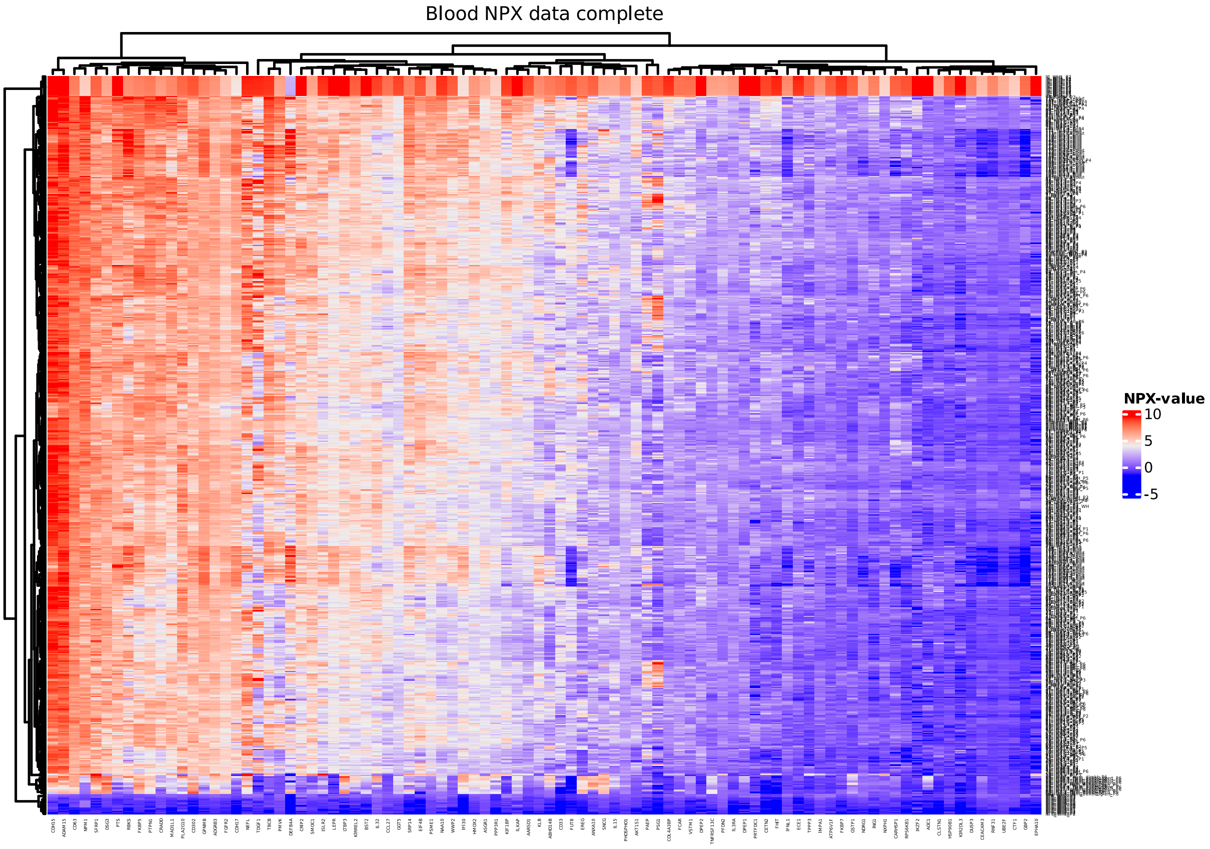

Figure S5: Heatmap based on the uncorrected NPX values from the complete set of serum samples (including positive and negative controls). The dendrogram in the heatmap was computed with the complete linkage method to find similar clusters based on the Euclidean distance.

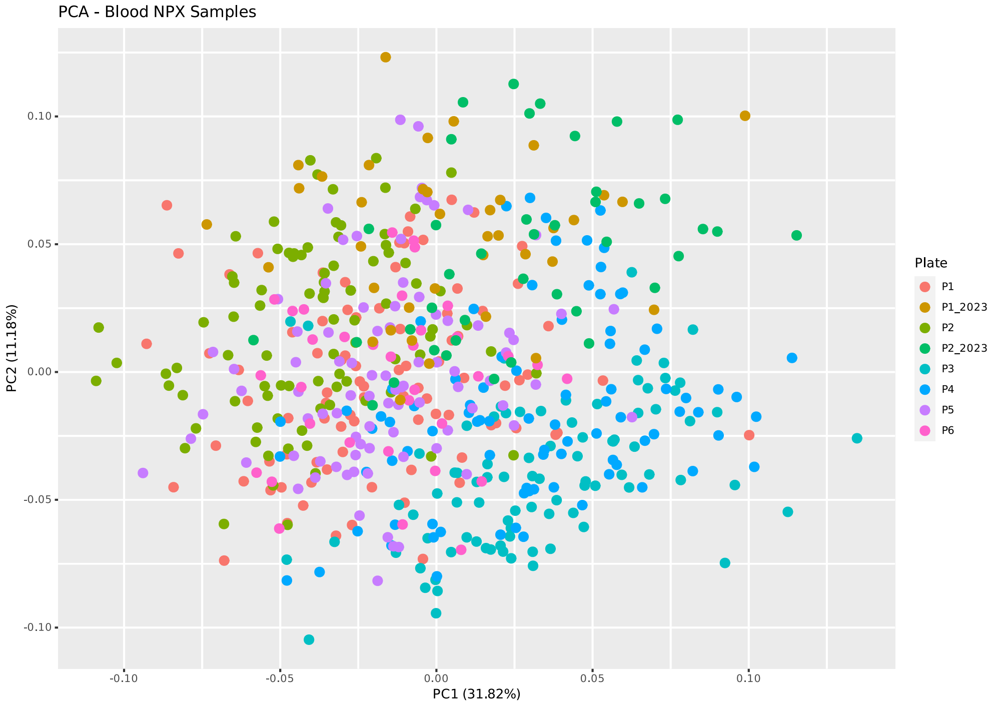

Figure S6: Score plot of the first two principal components based on a PCA performed on the complete set of uncorrected NPX-values of the serum samples. The percentage in the axis labels displays the percentage of the explained variance by this principal component. The dots are colored based on the corresponding Olink plate run.

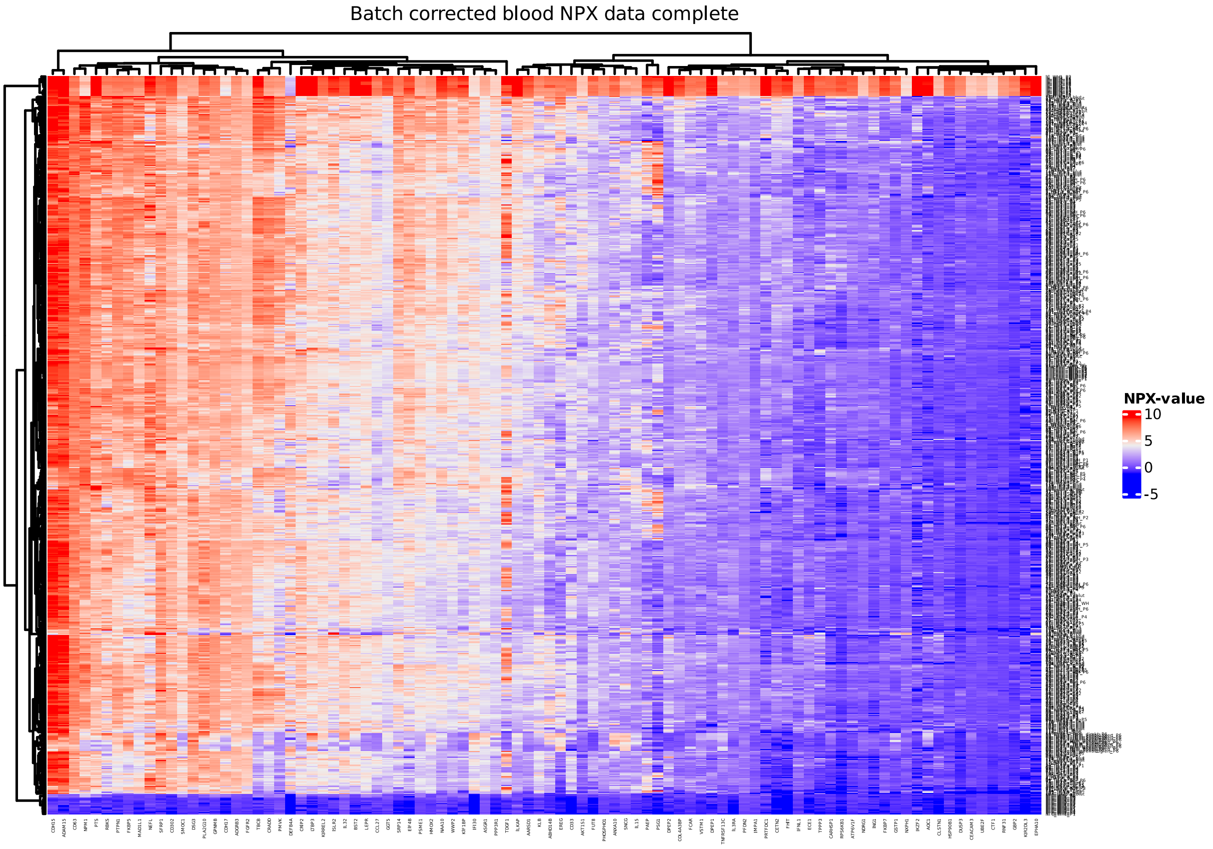

Figure S7: Heatmap based on the uncorrected NPX values from the complete set of serum samples (including positive and negative controls). The dendrogram in the heatmap was computed with the complete linkage method to find similar clusters based on the Euclidean distance.

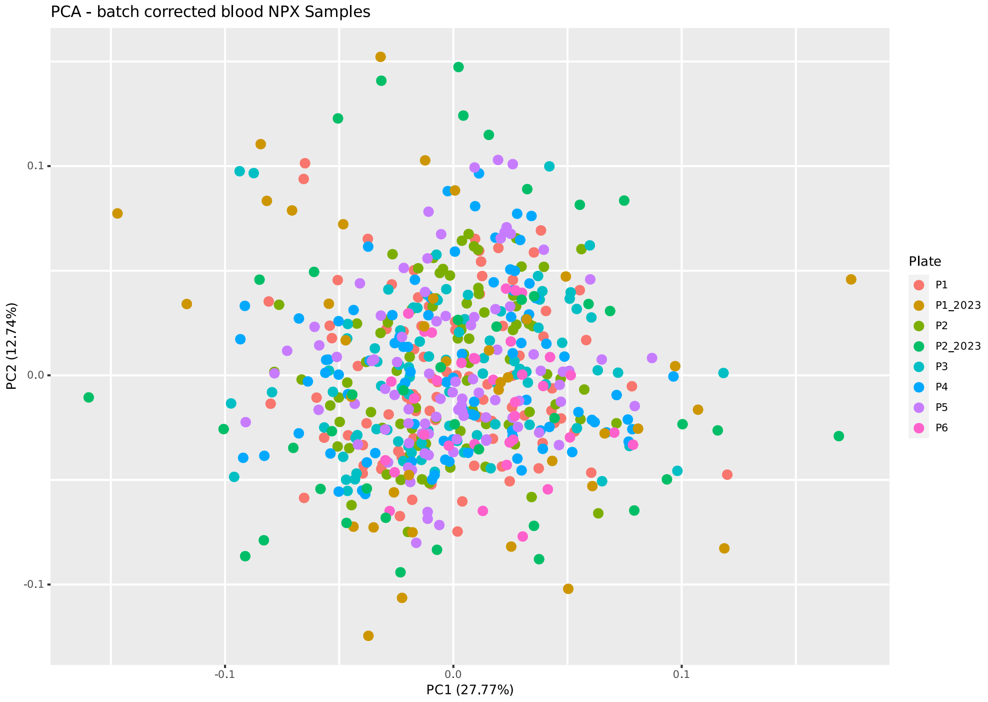

Figure S8: Score plot of the first two principal components based on a PCA performed on the complete set of uncorrected NPX-values of the serum samples. The percentage in the axis labels displays the percentage of the explained variance by this principal component. The dots are colored based on the corresponding Olink plate run.

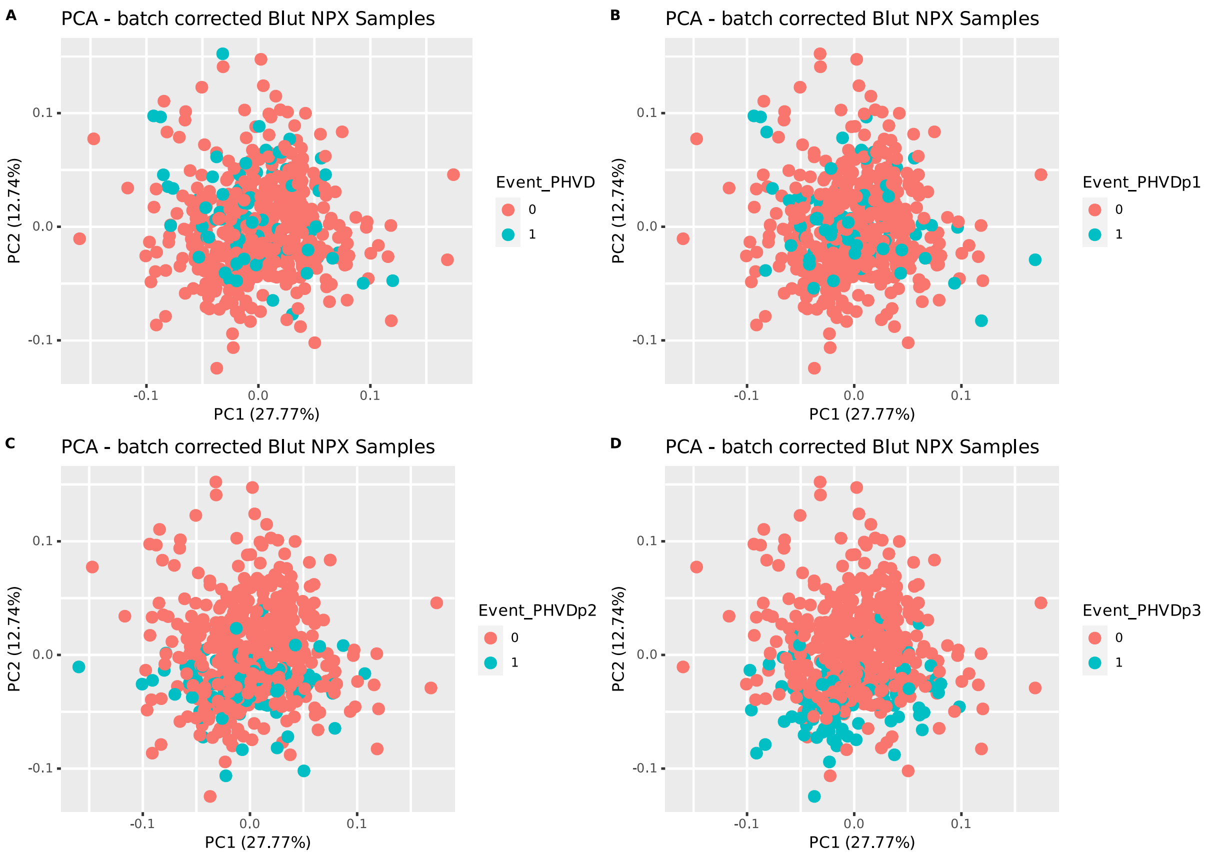

Figure S9: Four score plots of the first two principal components based on a PCA performed on the complete set of uncorrected NPX-values of the serum samples. The percentage in the axis labels displays the percentage of the explained variance by this principal component. The dots are colored in turquoise when being grouped in the time frames A – PHVD, B – PHVDp1, C – PHVDp2 and D – PHVDp3.

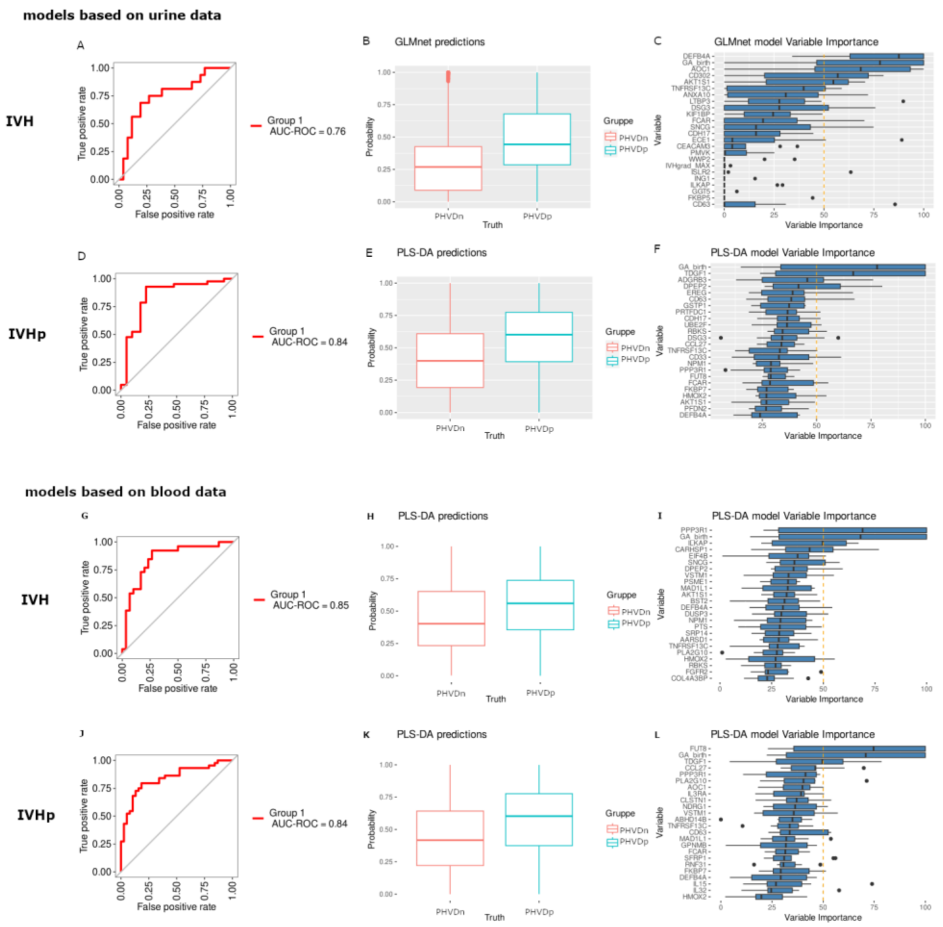

Figure S10: Overview of all best performing model types for the prediction of PHVD, based on urine and serum data separately. Each row of three plots describes a model trained on either urine or serum data from a specific timeframe. These three plots (A-C) give an overview of the 10 specific (GLMnet in this case) models trained on a specific timeframe (IVH urine) dataset for the prediction of PHVD. A, D, G, J – Area under the receiver operating curve (AUC-ROC). B, E, H, K– Box plot showing the predicted probability of developing a PHVD based on the test set for each model. C, F, I, L – horizontal boxplot indicating the variable importance of the 10 models, the threshold (50) is indicated by a vertical dotted orange line.

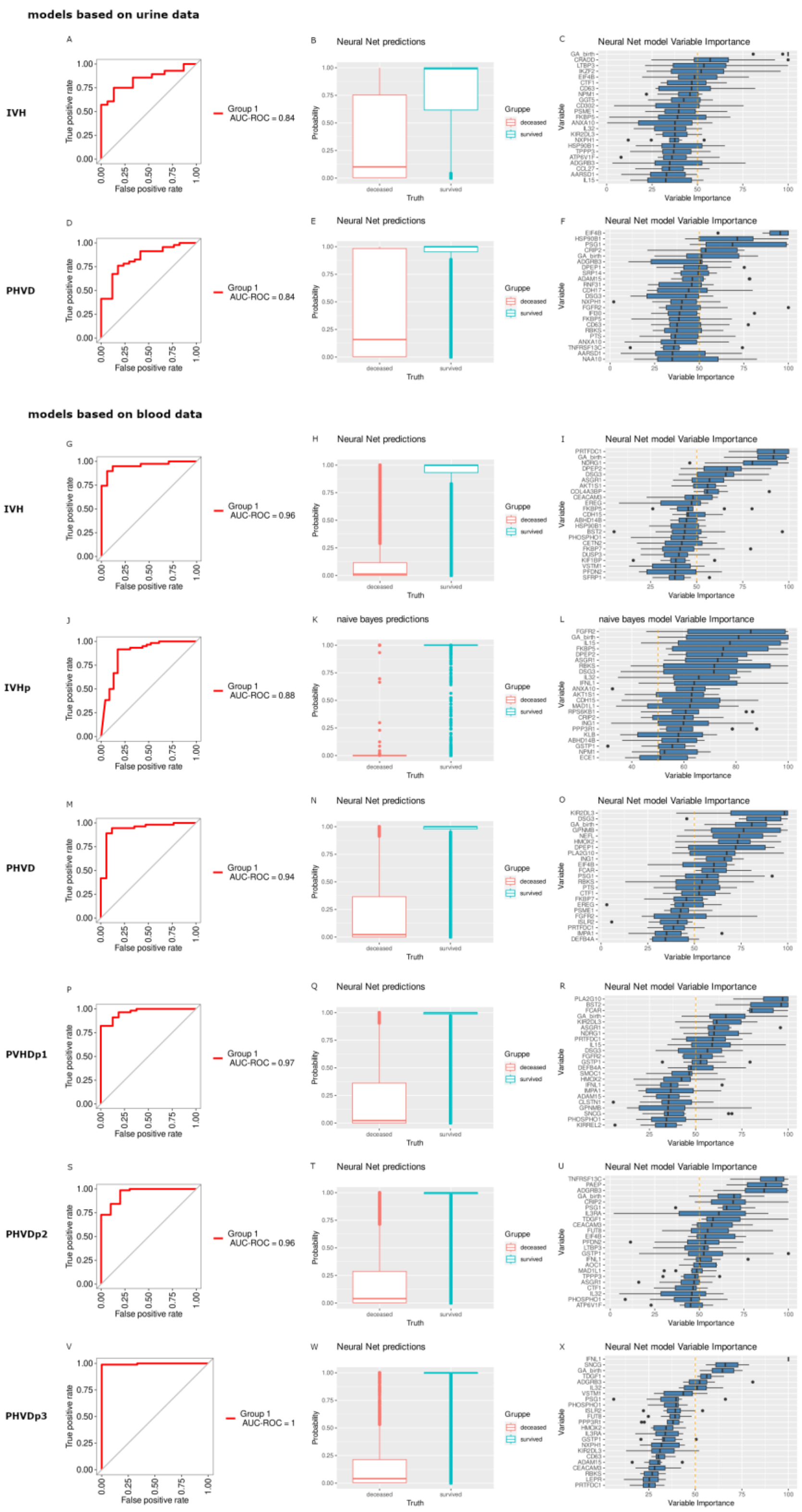

Figure S11: Overview of all best performing model types for the prediction of survival, based on urine and serum data separately. Each row of three plots describes a model trained on either urine or serum data from a specific timeframe. These three plots (A-C) give an overview of the 10 specific (neural network model in this case) models trained on a specific timeframe (IVH urine) dataset for the prediction of survival. A, D, G, J, H P, S, V – Area under the receiver operating curve (AUC-ROC). B, E, H, K, N, Q, T, W – Box plot showing the predicted probability of survival based on the test set for each model. C, F, I, L, O, R, U, X – horizontal boxplot indicating the variable importance of the 10 models, the threshold (50) is indicated by a vertical dotted orange line.

Table S1: Overview of urine samples. PHVD positive (PHVDp), PHVD negative (PHVDn) gestational age (GA), standard deviation (SD). The survival of the patients in the cohort is 70.7%, the % of survival displayed is related to the number of urine samples from patients. Intraventricular hemorrhage (IVH) grade was defined by Ultrasound analysis.

	**PHVDn** (n=194)	**PHVDp** (n=324)	**Total** (n=518)
**GA at birth**			
Mean (SD)	24.883 (1.735)	26.574 (2.168)	25.941 (2.175)
Range	23 – 29.714	23.286 – 33.286	23 – 33.286
**Survival**			
deceased	42 (21.6%)	55 (17.0%)	97 (18.7%)
survived	152 (78.4%)	269 (83.0%)	421 (81.3%)
**Sex**			
male	120 (61.9%)	230 (71.0%)	350 (67.6%)
female	74 (38.1%)	94 (29.0%)	168 (32.4%)
**IVHgrade_L**			
0	28 (14.4%)	4 (1.2%)	32 (6.2%)
2	49 (25.3%)	55 (17.0%)	104 (20.1%)
2.5	0 (0.0%)	4 (1.2%)	4 (0.8%)
3	56 (28.9%)	203 (62.7%)	259 (50.0%)
4	61 (31.4%)	58 (17.9%)	119 (23.0%)
**IVHgrade_R**			
0	2 (11.3%)	12 (3.7%)	4 (6.6%)
1	15 (7.7%)	4 (1.2%)	19 (3.7%)
2	58 (29.9%)	41 (12.7%)	99 (19.1%)
2.5	0 (0.0%)	4 (1.2%)	4 (0.8%)
3	68 (35.1%)	187 (57.7%)	255 (49.2%)
4	31 (16.0%)	76 (23.5%)	107 (20.7%)
**IVHuni_bi**			
uni-lateral	43 (22.2%)	16 (4.9%)	59 (11.4%)
bi-lateral	151 (77.8%)	308 (95.1%)	59 (88.6%)
**IVHgrade_MAX**			
2	48 (24.7%)	9 (2.8%)	57 (11.0%)
2.5	0 (0.0%)	4 (1.2%)	4 (0.8%)
3	64 (33.0%)	195 (60.2%)	259 (50.0%)
4	82 (42.3%)	116 (35.8%)	198 (38.2%)
**IVHgrade_SUM**			
2	26 (13.4%)	0 (0.0%)	26 (5.0%)
3	17 (8.8%)	16 (4.9%)	33 (6.4%)
4	32 (16.5%)	13 (4.0%)	45 (8.7%)
5	21 (10.8%)	33 (10.2%)	54 (10.4%)
6	63 (32.5%)	195 (60.2%)	258 (49.8%)
7	25 (12.9%)	49 (15.1%)	74 (14.3%)
8	10 (5.2%)	18 (5.6%)	28 (5.4%)

Table S2: Overview of serum samples. PHVD positive (PHVDp), PHVD negative (PHVDn) gestational age (GA), standard deviation (SD). The survival of the patients in the cohort is 70.7%, the % of survival displayed is related to the number of serum samples from patients. Intraventricular hemorrhage (IVH) grade was defined by Ultrasound analysis.

	**PHVDn** (n=224)	**PHVDp** (n=367)	**Total** (n=591)
**GA at birth**			
Mean (SD)	24.933 (1.713)	26.549 (2.136)	25.937 (2.134)
Range	23 – 29.714	23.286 – 33.286	23 – 33.286
**Survival**			
deceased	54 (24.1%)	62 (16.9%)	116 (19.6%)
survived	170 (75.9%)	305 (83.1%)	475 (80.4%)
**Sex**			
male	136 (60.7%)	267 (72.8%)	403 (68.2%)
female	88 (39.3%)	100 (27.2%)	188 (31.8%)
**IVHgrade_L**			
0	27 (12.1%)	9 (2.5%)	36 (6.1%)
2	58 (25.9%)	60 (16.3%)	118 (20.0%)
2.5	0 (0.0%)	5 (1.4%)	5 (0.8%)
3	76 (33.9%)	235 (64.0%)	311 (52.6%)
4	63 (28.1%)	58 (15.8%)	121 (20.5%)
**IVHgrade_R**			
0	31 (13.8%)	11 (3.0%)	42 (7.1%)
1	21 (9.4%)	4 (1.1%)	25 (4.2%)
2	60 (26.8%)	38 (10.4%)	98 (16.6%)
2.5	0 (0.0%)	5 (1.4%)	5 (0.8%)
3	75 (33.5%)	223 (60.8%)	298 (50.4%)
4	37 (16.5%)	86 (23.4%)	123 (20.8%)
**IVHuni_bi**			
uni-lateral	51 (22.8%)	20 (5.4%)	71 (12.0%)
bi-lateral	173 (77.2%)	347 (94.6%)	520 (88.0%)
**IVHgrade_MAX**			
2	50 (22.3%)	7 (1.9%)	57 (9.6%)
2.5	0 (0.0%)	5 (1.4%)	5 (0.8%)
3	86 (38.4%)	231 (62.9%)	317 (53.6%)
4	88 (39.3%)	124 (33.8%)	212 (35.9%)
**IVHgrade_SUM**			
2	25 (11.2%)	0 (0.0%)	25 (4.2%)
3	25 (11.2%)	20 (5.4%)	45 (7.6%)
4	41 (18.3%)	11 (3.0%)	52 (8.8%)
5	25 (11.2%)	36 (9.8%)	61 (10.3%)
6	72 (32.1%)	229 (62.4%)	301 (50.9%)
7	24 (10.7%)	51 (13.9%)	75 (12.7%)
8	12 (5.4%)	20 (5.4%)	32 (5.4%)

Table S3: Top 10 differentially expressed proteins comparing PHVDp vs. PHVDn patients in all possible comparisons based on the urine data. PHVD positive (PHVDp), PHVD negative (PHVDn). Group comparison using the limma R package [3] to identify significant changes in the NPX values.

Protein	Log2FC	Average NPX	*P* Value	Adjusted *P* Value
**Urine timepoint IVHp: PHVDp vs PHVDn**				
DPEP2	-0.4399	-0.7286	0.0142	0.5648
PRTFDC1	-0.5193	-0.6210	0.0231	0.5648
FCAR	-0.5809	0.3556	0.0266	0.5648
PAEP	0.4732	-0.2529	0.0269	0.5648
TDGF1	0.3865	-0.3621	0.0335	0.5648
CCL27	-0.2645	-0.2801	0.0368	0.5648
CEACAM3	-0.156	-0.3402	0.1147	0.9582
PHOSPHO1	0.2378	0.4659	0.1284	0.9582
ECE1	0.2383	-0.8693	0.1302	0.9582
PSG1	0.4304	-0.4306	0.1383	0.9582
**Urine timepoint PHVD: PHVDp vs PHVDn**				
KIRREL2	1.7960	0.8471	0.0749	0.9990
EIF4B	1.5421	-0.5512	0.0809	0.9990
TBCB	0.9933	-0.5954	0.0854	0.9990
CD302	-2.5512	3.8481	0.0897	0.9990
CDH15	2.7972	3.6693	0.0925	0.9990
NEFL	1.9738	1.5678	0.0977	0.9990
DEFB4A	2.4940	-1.0650	0.1262	0.9990
FHIT	0.9664	-1.6331	0.1341	0.9990
CEACAM3	-0.4354	-0.3402	0.2106	0.9990
NXPH1	0.5494	-0.0350	0.2132	0.9990
**Urine timepoint PHVDp3: PHVDp vs PHVDn**				
ADGRB3	-2.3291	3.2765	0.0028	0.1326
EPHA10	-2.6648	-0.4189	0.0038	0.1326
CD302	-4.1787	3.8481	0.0060	0.1326
ADAM15	-2.6937	3.2036	0.0068	0.1326
ASGR1	-2.1191	2.3090	0.0072	0.1326
CDH17	-1.8628	-0.3733	0.0106	0.1537
SFRP1	-4.1222	4.1955	0.0144	0.1537
KIR2DL3	-1.6150	-1.1862	0.0147	0.1537
LTBP3	-3.0741	3.9389	0.0166	0.1537
TDGF1	1.5441	-0.3621	0.0167	0.1537
ADGRB3	-2.3291	3.2765	0.0028	0.1326
**Urine timepoint 32 weeks GA: PHVDp vs PHVDn**				
ADAM15	2.7955	3.2036	0.0002	0.0184
ADGRB3	1.8179	3.2765	0.0019	0.0607
CDH17	1.6974	-0.3733	0.0019	0.0607
CD302	3.1045	3.8481	0.0065	0.1209
ASGR1	1.6034	2.3090	0.0067	0.1209
LTBP3	2.5310	3.9389	0.0086	0.1209
BST2	1.7539	1.3192	0.0092	0.1209
CD63	2.2053	3.4013	0.0115	0.1290
SFRP1	3.1501	4.1955	0.0127	0.1290
PLA2G10	2.2165	1.2865	0.0150	0.1290

Table S4: Top 10 differentially expressed proteins between different timepoints based on the urine data. Group comparison using the *limma* R package [3] to identify significant changes in the NPX values.

Protein	Log2FC	Average NPX	*P* Value	Adjusted *P* Value
**Urine: IVH vs IVHp**				
PAEP	1.3696	-0.1560	1.3081 e^{-21}	1.2035 e^{-19}
PSG1	1.4568	-0.4117	4.4984 e^{-14}	2.0693 e^{-12}
CRADD	0.7423	0.1660	8.4364 e^{-10}	2.5871 e^{-8}
FHIT	0.6478	-1.3824	6.4854 e^{-7}	1.4916 e^{-5}
NXPH1	0.3981	0.0526	8.4122 e^{-6}	0.0001
EIF4B	0.7049	-0.3578	8.1777 e^{-5}	0.0012
ABHD14B	0.6732	0.4217	0.0001	0.0012
CD33	0.5803	-1.0977	0.0001	0.0012
CDH15	1.2552	3.5429	0.0001	0.0015
PFDN2	0.2081	0.2783	0.0014	0.0118
**Urine: IVH vs PHVD**				
EREG	-1.6462	2.0361	0.0318	0.9979
IFI30	-1.1019	4.1713	0.0790	0.9979
NDRG1	0.5557	-0.0773	0.0962	0.9979
ABHD14B	-0.9344	0.4217	0.1322	0.9979
CD33	-0.7914	-1.0977	0.1415	0.9979
PRTFDC1	-0.8102	-0.3546	0.1715	0.9979
FHIT	-0.6023	-1.3824	0.1947	0.9979
SFRP1	-1.5160	4.0590	0.2106	0.9979
SMOC1	-0.5683	0.4274	0.2118	0.9979
PAEP	-0.6178	-0.1560	0.2130	0.9979
**Urine: IVH vs PHVDp1**				
EREG	-1.9204	2.0361	0.0088	0.8125
IFI30	-1.2006	4.1713	0.0451	0.9701
ABHD14B	-1.0162	0.4217	0.0865	0.9701
NDRG1	0.5329	-0.0773	0.0948	0.9701
NPM1	-1.4982	1.6128	0.1194	0.9701
SNCG	1.2447	2.3998	0.1388	0.9701
PAEP	-0.6737	-0.1560	0.1550	0.9701
COL4A3BP	-0.3982	-0.4002	0.1608	0.9701
SFRP1	-1.5958	4.0590	0.1676	0.9701
CD63	-1.0556	3.3731	0.1777	0.9701
EREG	-1.9204	2.0361	0.0088	0.9701
**Urine: IVH vs PHVDp2**				
EREG	-2.1087	2.0361	0.0054	0.4971
IFI30	-1.5322	4.1713	0.0134	0.6180
ABHD14B	-1.3963	0.4217	0.0227	0.6988
SFRP1	-2.3428	4.0590	0.0501	0.7273
NPM1	-1.9396	1.6128	0.0512	0.7273
TBCB	-0.6893	-0.3802	0.0756	0.7273
ASGR1	-0.9502	2.3924	0.0877	0.7273
CD33	-0.9050	-1.0977	0.0885	0.7273
TNFRSF13C	-0.9581	2.2143	0.1078	0.7273
MAD1L1	-1.1543	1.2511	0.1101	0.7273

Protein	Log2FC	Average NPX	*P* Value	Adjusted *P* Value
**Urine: IVH vs PHVDp3**				
EREG	-2.2692	2.0361	0.0030	0.2561
ABHD14B	-1.7210	0.4217	0.0055	0.2561
IFI30	-1.4838	4.1713	0.0178	0.5206
SFRP1	-2.6612	4.0590	0.0278	0.5206
CD33	-1.1794	-1.0977	0.0282	0.5206
ASGR1	-1.1672	2.3924	0.0381	0.5444
CDH15	-2.4039	3.5429	0.0424	0.5444
AOC1	-1.5864	0.5662	0.0549	0.5444
EPHA10	-1.2673	-0.2115	0.0606	0.5444
ECE1	-0.6776	-0.6447	0.0749	0.5444
**Urine: IVHp vs PHVD**				
PAEP	-1.9874	-0.1560	0.0001	0.0095
FHIT	-1.2502	-1.3824	0.0088	0.2425
CRADD	-1.1221	0.1660	0.0109	0.2425
ABHD14B	-1.6076	0.4217	0.0117	0.2425
CD33	-1.3717	-1.0977	0.0131	0.2425
SFRP1	-2.4233	4.0590	0.0513	0.7128
CDH15	-2.2788	3.5429	0.0613	0.7128
IFI30	-1.2016	4.1713	0.0619	0.7128
TBCB	-0.6966	-0.3802	0.0842	0.7154
EPHA10	-1.1751	-0.2115	0.0906	0.7154
**Urine: IVHp vs PHVDp1**				
PAEP	-2.0433	-0.1560	3.0395 e^{-5}	0.0027
ABHD14B	-1.6895	0.4217	0.0056	0.2585
CRADD	-1.1048	0.1660	0.0087	0.2695
CD33	-1.2171	-1.0977	0.0213	0.4633
FHIT	-0.9783	-1.3824	0.0319	0.4633
IFI30	-1.3003	4.1713	0.0346	0.4633
SFRP1	-2.5031	4.0590	0.0352	0.4633
CDH15	-2.3375	3.5429	0.0446	0.4918
EREG	-1.4787	2.0361	0.0492	0.4918
NPM1	-1.9090	1.6128	0.0534	0.4918
**Urine: IVHp vs PHVDp2**				
PAEP	-2.0415	-0.1560	5.2586 e^{-5}	0.0048
ABHD14B	-2.0695	0.4217	0.0010	0.0467
CRADD	-1.3242	0.1660	0.0023	0.0717
CD33	-1.4853	-1.0977	0.0064	0.1327
SFRP1	-3.2500	4.0590	0.0080	0.1327
FHIT	-1.2355	-1.3824	0.0086	0.1327
IFI30	-1.6318	4.1713	0.0101	0.1338
CDH15	-3.0067	3.5429	0.0123	0.1417
NPM1	-2.3504	1.6128	0.0211	0.2140
TBCB	-0.9028	-0.3802	0.0232	0.2140

**Protein**	**Log2FC**	**Average NPX**	***P* Value**	**Adjusted *P* Value**
**Urine: IVHp vs PHVDp3**				
PAEP	-2.1460	-0.1560	2.4976 e^{-5}	0.0022
ABHD14B	-2.3943	0.4217	0.0001	0.0076
CRADD	-1.4418	0.1660	0.0010	0.0312
CD33	-1.7598	-1.0977	0.0013	0.0319
CDH15	-3.6592	3.5429	0.0025	0.0468
FHIT	-1.3700	-1.3824	0.0038	0.0516
SFRP1	-3.5685	4.0590	0.0039	0.0516
EPHA10	-1.7169	-0.2115	0.0128	0.1364
IFI30	-1.5834	4.1713	0.0133	0.1364
EREG	-1.8274	2.0361	0.0194	0.1785
**Urine: PHVD vs PHVDp1**				
ISLR2	0.4858	1.1267	0.0144	0.7017
DEFB4A	0.7454	-1.0093	0.0377	0.7017
IL32	-0.3296	0.7396	0.0429	0.7017
HSP90B1	0.2181	-0.3994	0.0448	0.7017
FHIT	0.2718	-1.3824	0.0517	0.7017
LTBP3	-0.5581	3.8701	0.0524	0.7017
ADAM15	-0.4047	3.2300	0.0552	0.7017
FUT8	0.2151	-0.9847	0.0664	0.7017
TNFRSF13C	-0.3233	2.2143	0.0749	0.7017
IFNL1	0.1365	-0.0533	0.1051	0.7017
**Urine: PHVD vs PHVDp2**				
TNFRSF13C	-0.7325	2.2143	6.5986 e^{-5}	0.0060
CDH17	-0.5289	-0.3088	0.0003	0.0169
ADAM15	-0.7087	3.2300	0.0008	0.0179
IL32	-0.5463	0.7396	0.0008	0.0179
FGFR2	-0.5518	2.7377	0.0010	0.0179
LTBP3	-0.9368	3.8701	0.0012	0.0179
AARSD1	-0.5772	-0.3452	0.0013	0.0179
RBKS	-0.9300	1.6521	0.0021	0.0244
DEFB4A	1.0799	-1.0093	0.0028	0.0287
ASGR1	-0.4951	2.3924	0.0037	0.0345
**Urine: PHVD vs PHVDp3**				
IKZF2	-1.1524	0.3549	0.0004	0.0144
ABHD14B	-0.7866	0.4217	0.0004	0.0144
ASGR1	-0.7121	2.3924	0.0004	0.0144
FGFR2	-0.6590	2.7377	0.0010	0.0214
CDH15	-1.3804	3.5429	0.0012	0.0214
AARSD1	-0.6851	-0.3452	0.0014	0.0214
TNFRSF13C	-0.6737	2.2143	0.0019	0.0259
IL32	-0.5705	0.7396	0.0034	0.0398
CDH17	-0.4879	-0.3088	0.0056	0.0577
EIF4B	-0.6242	-0.3578	0.0069	0.0619

**Protein**	**Log2FC**	**Average NPX**	***P* Value**	**Adjusted *P* Value**
**Urine: PHVDp1 vs PHVDp2**				
KLB	-0.2903	-0.0984	0.0005	0.0221
IKZF2	-0.8227	0.3549	0.0008	0.0221
TBCB	-0.3535	-0.3802	0.0008	0.0221
ECE1	-0.3403	-0.6447	0.0009	0.0221
AKT1S1	-0.2345	-0.5533	0.0016	0.0300
EIF4B	-0.5218	-0.3578	0.0026	0.0352
RBKS	-0.8016	1.6521	0.0029	0.0352
NXPH1	-0.2557	0.0526	0.0030	0.0352
MAD1L1	-0.5355	1.2511	0.0067	0.0691
GPNMB	-0.4136	1.5035	0.0113	0.0997
**Urine: PHVDp1 vs PHVDp3**				
IKZF2	-1.1935	0.3549	0.0001	0.0144
NXPH1	-0.4029	0.0526	0.0003	0.0144
ECE1	-0.4425	-0.6447	0.0008	0.0262
ABHD14B	-0.7047	0.4217	0.0011	0.0262
CDH15	-1.3217	3.5429	0.0014	0.0262
HSP90B1	-0.3796	-0.3994	0.0026	0.0402
EIF4B	-0.6468	-0.3578	0.0038	0.0411
CD33	-0.5426	-1.0977	0.0038	0.0411
ASGR1	-0.5528	2.3924	0.0049	0.0411
KIRREL2	-0.6969	0.9232	0.0051	0.0411
**Urine: PHVDp2 vs PHVDp3**				
CD63	0.4648	3.3731	0.0616	0.7985
NEFL	-0.4968	1.8308	0.0683	0.7985
CDH15	-0.6524	3.5429	0.0697	0.7985
NDRG1	0.1805	-0.0773	0.0746	0.7985
ABHD14B	-0.3247	0.4217	0.0844	0.7985
CD33	-0.2744	-1.0977	0.0926	0.7985
CARHSP1	0.1912	-0.2598	0.1027	0.7985
CRIP2	0.2599	0.2043	0.1039	0.7985
AKT1S1	0.1346	-0.5533	0.1067	0.7985
GGT5	-0.2720	0.7462	0.1090	0.7985
**Urine: IVH & IVHp vs 28 days of life**				
PAEP	2.4471	-0.1560	$1.2315\ e^{-6}$	0.0001
CRADD	1.2486	0.1660	0.0039	0.1817
ABHD14B	1.6448	0.4217	0.0086	0.2645
FHIT	1.1584	-1.3824	0.0134	0.3088
CD33	1.2299	-1.0977	0.0234	0.4313
EREG	1.6049	2.0361	0.0376	0.5776
CD63	1.5856	3.3731	0.0549	0.6269
TBCB	0.7561	-0.3802	0.0562	0.6269
PRTFDC1	1.1164	-0.3546	0.0614	0.6269
SFRP1	2.1737	4.0590	0.0748	0.6269

**Protein**	**Log2FC**	**Average NPX**	***P* Value**	**Adjusted *P* Value**
**Urine: IVH & IVHp vs 32 weeks GA**				
PAEP	2.3665	-0.1560	$4.0216\ e^{-6}$	0.0003
CRADD	1.3772	0.1660	0.0018	0.0834
ABHD14B	1.5445	0.4217	0.0154	0.4002
FHIT	1.1347	-1.3824	0.0174	0.4002
CD33	1.2393	-1.0977	0.0249	0.4592
EIF4B	1.3399	-0.3578	0.0417	0.5668
SMOC1	0.9411	0.4274	0.0440	0.5668
PRTFDC1	1.1955	-0.3546	0.0492	0.5668
CD63	1.4072	3.3731	0.0943	0.7823
LTBP3	-1.6385	3.8701	0.0950	0.7823
**Urine: IVH & IVHp vs Term equivalent age**				
PAEP	2.3429	-0.1560	$8.2046\ e^{-6}$	0.0007
ABHD14B	1.6871	0.4217	0.0097	0.3922
CD33	1.3678	-1.0977	0.0157	0.3922
CRADD	1.0766	0.1660	0.0170	0.3922
PRTFDC1	1.4098	-0.3546	0.0236	0.4208
EREG	1.7766	2.0361	0.0274	0.4208
FHIT	1.0352	-1.3824	0.0340	0.4473
SFRP1	2.5488	4.0590	0.0452	0.5206
CD63	1.6181	3.3731	0.0604	0.6178
PSG1	1.2515	-0.4117	0.0792	0.6308
**Urine: 28 days of life vs 32 weeks GA**				
FGFR2	-0.5916	2.7377	0.0007	0.0279
LTBP3	-1.0100	3.8701	0.0007	0.0279
TNFRSF13C	-0.6285	2.2143	0.0009	0.0279
SRP14	-0.7600	0.7275	0.0071	0.1639
LEPR	-0.2755	-0.4448	0.0186	0.2974
CEACAM3	-0.1733	-0.1994	0.0237	0.2974
HSP90B1	0.2532	-0.3994	0.0251	0.2974
RPS6KB1	-0.2315	-0.6453	0.0276	0.2974
CD302	-0.7294	3.9514	0.0318	0.2974
BST2	-0.3999	1.4251	0.0323	0.2974
**Urine: 28 days of life vs Term equivalent age**				
CDH17	0.5219	-0.3088	0.0024	0.2278
PLA2G10	0.7082	1.3484	0.0191	0.6035
GSTP1	-0.4661	0.9024	0.0259	0.6035
PFDN2	-0.1808	0.2783	0.0282	0.6035
CD302	0.7697	3.9514	0.0439	0.6035
NAA10	-0.3367	0.8739	0.0491	0.6035
NDRG1	-0.2212	-0.0773	0.0595	0.6035
FKBP5	-0.6166	3.1580	0.0616	0.6035
CARHSP1	-0.2463	-0.2598	0.0699	0.6035
DSG3	-0.5496	4.5903	0.0770	0.6035

Protein	Log2FC	Average NPX	*P* Value	Adjusted *P* Value
**Urine: 32 weeks GA vs Term equivalent age**				
LTBP3	1.4684	3.8701	7.2215 e^{-6}	0.0006
CD302	1.4992	3.9514	5.2853 e^{-5}	0.0024
BST2	0.7333	1.4251	0.0003	0.0076
ADAM15	0.8537	3.2300	0.0003	0.0076
ASGR1	0.6783	2.3924	0.0004	0.0076
FGFR2	0.6351	2.7377	0.0008	0.0126
IL3RA	0.3584	-0.5431	0.0025	0.0334
ADGRB3	0.5771	3.2601	0.0032	0.0368
CDH15	1.1022	3.5429	0.0062	0.0642
GPNMB	0.5432	1.5035	0.0083	0.0765

Table S5: Top 10 differentially expressed proteins comparing PHVDp vs. PHVDn patients in all possible comparisons based on the serum data.
PHVD positive (PHVDp), PHVD negative (PHVDn). Group comparison using the *limma* R package[3] to identify significant changes in the NPX values.

Protein	Log2FC	Average NPX	*P* Value	Adjusted *P* Value
**Serum timepoint IVHp: PHVDp vs PHVDn**				
AOC1	-0.6187	0.2219	0.0032	0.2666
FUT8	0.5440	2.6107	0.0090	0.2666
IL3RA	-0.3344	1.4136	0.0099	0.2666
CD33	-0.6037	3.0904	0.0138	0.2666
LEPR	-0.4457	4.2291	0.0144	0.2666
NEFL	0.6139	5.7979	0.0237	0.2934
SNCG	0.5251	2.4993	0.0243	0.2934
NXPH1	0.3632	0.6565	0.0255	0.2934
SFRP1	0.5066	6.3729	0.0413	0.4223
PFDN2	0.3255	1.5841	0.0560	0.4684
**Serum timepoint PHVD: PHVDp vs PHVDn**				
KIRREL2	2.4498	4.5185	0.0645	0.9988
HSP90B1	2.2385	0.0249	0.0754	0.9988
CDH15	2.6502	8.6450	0.0993	0.9988
FCAR	-3.1662	1.9594	0.1103	0.9988
EPHA10	-2.9734	-0.3913	0.1432	0.9988
IFNL1	2.7840	1.0111	0.1509	0.9988
FKBP5	-3.3945	5.6713	0.1587	0.9988
DSG3	2.3600	6.1418	0.1885	0.9988
VSTM1	-2.7436	1.7269	0.1971	0.9988
CRIP2	-2.4828	4.9724	0.2221	0.9988
**Serum timepoint PHVDp2: PHVDp vs PHVDn**				
EPHA10	-2.7400	-0.3913	0.0552	0.9756
VSTM1	-2.7692	1.7269	0.0643	0.9756
FCAR	-2.2337	1.9594	0.1086	0.9756
FKBP5	-2.4437	5.6713	0.1484	0.9756
KIR2DL3	-1.3875	-0.1644	0.1848	0.9756
TPPP3	-1.9862	0.6934	0.2016	0.9756
CD302	-1.1723	5.6742	0.2110	0.9756
EIF4B	-1.9783	4.7028	0.2296	0.9756
CD63	1.0614	7.1784	0.2422	0.9756
IFNL1	-1.5419	1.0111	0.2567	0.9756
**Serum timepoint PHVDp3: PHVDp vs PHVDn**				
KIR2DL3	-3.2259	-0.1644	0.0129	0.5476
HMOX2	3.7731	4.1179	0.0147	0.5476
MAD1L1	4.4251	5.6203	0.0178	0.5476
CD302	-2.4599	5.6742	0.0339	0.6938
TDGF1	-7.4418	4.6362	0.0402	0.6938
PSME1	3.5691	4.5255	0.0475	0.6938
COL4A3BP	3.1162	1.9969	0.0565	0.6938
DPEP1	2.0740	1.3472	0.0636	0.6938
DPEP2	2.5004	1.7757	0.0757	0.6938
SRP14	3.8349	4.8067	0.0807	0.6938

Protein	Log2FC	Average NPX	*P* Value	Adjusted *P* Value
**Serum timepoint 32 weeks GA: PHVDp vs PHVDn**				
KIR2DL3	1.9913	-0.1644	0.0739	0.9973
CD63	-1.3573	7.1784	0.1598	0.9973
CD302	1.2941	5.6742	0.1940	0.9973
TDGF1	3.6400	4.6362	0.2426	0.9973
FUT8	-1.3782	2.6107	0.2705	0.9973
KIRREL2	-1.0457	4.5185	0.2888	0.9973
EPHA10	1.6050	-0.3913	0.2889	0.9973
VSTM1	1.6756	1.7266	0.2908	0.9973
FCAR	1.5456	1.9594	0.2952	0.9973
TPPP3	1.7273	0.6934	0.2958	0.9973

Table S6: Top 10 differentially expressed proteins between different timepoints based on the serum data. Group comparison using the *limma* R package [3] to identify significant changes in the NPX values.

Protein	Log2FC	Average NPX	*P* Value	Adjusted *P* Value
**Serum: IVH vs IVHp**				
PSG1	2.0897	2.3164	2.8927 e^{-6}	0.0002
PAEP	1.2720	2.4293	0.0015	0.0726
NXPH1	0.5515	0.6700	0.0081	0.2489
NEFL	-0.6697	5.7718	0.0610	0.9790
PMVK	-0.6783	4.6089	0.0719	0.9790
KLB	0.4700	3.3328	0.1056	0.9790
CRIP2	0.5523	4.9117	0.1476	0.9790
DSG3	0.4245	5.9457	0.1517	0.9790
IFI30	-0.3748	4.0460	0.1964	0.9790
TNFRSF13C	0.2967	1.5303	0.2212	0.9790
**Serum: IVH vs PHVD**				
PSG1	2.3330	2.3164	0.0364	0.9913
DSG3	-0.9369	5.9457	0.2098	0.9913
FKBP5	0.7429	5.6880	0.3883	0.9913
PTS	-0.8124	5.9466	0.3926	0.9913
IL15	0.4561	2.4785	0.4590	0.9913
CRADD	0.6073	5.4249	0.4712	0.9913
DEFB4A	-0.6238	4.4902	0.4891	0.9913
TBCB	0.6699	5.4333	0.5323	0.9913
ADGRB3	-0.3755	5.4937	0.5483	0.9913
PFDN2	0.3339	1.6456	0.5759	0.9913
**Serum: IVH vs PHVDp1**				
PSG1	3.6169	2.3164	0.0003	0.0316
DSG3	-1.2153	5.9457	0.0714	0.9967
IL15	0.8181	2.4785	0.1410	0.9967
FKBP5	0.9560	5.6880	0.2183	0.9967
CRADD	0.8106	5.4249	0.2862	0.9967
IFI30	-0.5665	4.0460	0.3905	0.9967
ANXA10	-0.6307	2.6370	0.3964	0.9967
LTBP3	-0.6497	4.5236	0.4315	0.9967
NXPH1	0.3655	0.6700	0.4388	0.9967
KLB	0.5087	3.3328	0.4409	0.9967
**Serum: IVH vs PHVDp2**				
PSG1	3.0100	2.3164	0.0043	0.4004
DSG3	-1.2073	5.9457	0.0873	0.9931
IL15	0.7596	2.4785	0.1919	0.9931
KLB	0.8077	3.3328	0.2430	0.9931
CD302	0.8704	5.5844	0.2640	0.9931
NEFL	0.8940	5.7718	0.2934	0.9931
CD33	-0.8121	3.0243	0.3296	0.9931
IL32	-0.6814	4.2740	0.3332	0.9931
RBKS	-0.8351	5.6700	0.3354	0.9931
IFI30	-0.6595	4.0460	0.3400	0.9931

**Protein**	**Log2FC**	**Average NPX**	***P* Value**	**Adjusted *P* Value**
**Serum: IVH vs PHVDp3**				
PSG1	3.6396	2.3164	0.0013	0.1218
DSG3	-1.1895	5.9457	0.1161	0.9907
IL15	0.8615	2.4785	0.1676	0.9907
NEFL	1.2403	5.7718	0.1743	0.9907
VSTM1	1.0089	1.8421	0.2136	0.9907
CD302	0.9841	5.5844	0.2390	0.9907
IFI30	-0.7909	4.0460	0.2861	0.9907
KLB	0.7129	3.3328	0.3365	0.9907
SMOC1	0.5776	4.8257	0.3632	0.9907
DEFB4A	0.8223	4.4902	0.3681	0.9907
**Serum: IVHp vs PHVD**				
DSG3	-1.3614	5.9457	0.0822	0.9963
PAEP	-1.2845	2.4293	0.2251	0.9963
FKBP5	1.0042	5.6880	0.2658	0.9963
CRADD	0.9412	5.4249	0.2867	0.9963
PMVK	1.0211	4.6089	0.3048	0.9963
PTS	-1.0128	5.9466	0.3092	0.9963
CARHSP1	0.8467	0.9468	0.3483	0.9963
TBCB	0.9988	5.4333	0.3743	0.9963
ISLR2	-0.7802	4.3091	0.3872	0.9963
NXPH1	-0.4736	0.6700	0.3881	0.9963
**Serum: IVHp vs PHVDp1**				
DSG3	-1.6398	5.9457	0.0213	0.9965
FKBP5	1.2174	5.6880	0.1377	0.9965
PSG1	1.5271	2.3164	0.1495	0.9965
CRADD	1.1444	5.4249	0.1540	0.9965
IL15	0.7515	2.4785	0.2001	0.9965
PMVK	1.1450	4.6089	0.2053	0.9965
PAEP	-1.0374	2.4293	0.2806	0.9965
CARHSP1	0.8308	0.9468	0.3109	0.9965
TBCB	0.9805	5.4333	0.3370	0.9965
AKT1S1	0.6873	2.5422	0.3376	0.9965
**Serum: IVHp vs PHVDp2**				
DSG3	-1.6318	5.9457	0.0286	0.9818
NEFL	1.5637	5.7718	0.0818	0.9818
IL32	-0.9509	4.2740	0.2005	0.9818
VSTM1	0.9414	1.8421	0.2379	0.9818
CD302	0.9402	5.5844	0.2526	0.9818
ISLR2	-0.9716	4.3091	0.2576	0.9818
IL15	0.6930	2.4785	0.2589	0.9818
CDH15	-1.0088	8.3480	0.2723	0.9818
PTS	-1.0371	5.9466	0.2735	0.9818
CARHSP1	0.8776	0.9468	0.3066	0.9818

Protein	Log2FC	Average NPX	*P* Value	Adjusted *P* Value
**Serum: IVHp vs PHVDp3**				
DSG3	-1.6141	5.9457	0.0423	0.9939
NEFL	1.9100	5.7718	0.0463	0.9939
VSTM1	1.2856	1.8421	0.1310	0.9939
PSG1	1.5499	2.3164	0.1900	0.9939
IL15	0.7949	2.4785	0.2247	0.9939
CD302	1.0539	5.5844	0.2293	0.9939
TNFRSF13C	-0.7719	1.5303	0.2353	0.9939
IL32	-0.8805	4.2740	0.2663	0.9939
ISLR2	-0.9879	4.3091	0.2804	0.9939
DEFB4A	1.0285	4.4902	0.2833	0.9939
**Serum: PHVD vs PHVDp1**				
PSG1	1.2839	2.3164	0.0206	0.9892
ISLR2	0.5720	4.3091	0.1818	0.9892
DEFB4A	0.5335	4.4902	0.2343	0.9892
IL15	0.3619	2.4785	0.2374	0.9892
NXPH1	0.2875	0.6700	0.2695	0.9892
AKT1S1	0.3463	2.5422	0.3551	0.9892
ANXA10	-0.3615	2.6370	0.3781	0.9892
HSP90B1	0.3388	0.2026	0.3993	0.9892
CCL27	0.2857	3.5706	0.4394	0.9892
CDH17	0.2501	5.2892	0.4475	0.9892
**Serum: PHVD vs PHVDp2**				
NEFL	1.2217	5.7718	0.0071	0.6571
IL32	-0.7683	4.2740	0.0405	0.9773
VSTM1	0.7903	1.8421	0.0497	0.9773
RBKS	-0.8995	5.6700	0.0514	0.9773
PMVK	-0.8022	4.6089	0.0929	0.9773
KLB	0.5635	3.3328	0.1257	0.9773
PAEP	0.7487	2.4293	0.1403	0.9773
SMOC1	0.4252	4.8257	0.1773	0.9773
TBCB	-0.6614	5.4333	0.2199	0.9773
PSG1	0.6770	2.3164	0.2257	0.9773
**Serum: PHVD vs PHVDp3**				
NEFL	1.5680	5.7718	0.0063	0.5499
DEFB4A	1.4462	4.4902	0.0119	0.5499
VSTM1	1.1345	1.8421	0.0261	0.8024
PSG1	1.3066	2.3164	0.0650	0.9973
IL32	-0.6979	4.2740	0.1410	0.9973
SMOC1	0.5611	4.8257	0.1595	0.9973
HMOX2	-0.5436	4.0660	0.2079	0.9973
CD302	0.5963	5.5844	0.2555	0.9973
IL15	0.4054	2.4785	0.3005	0.9973
KLB	0.4687	3.3328	0.3139	0.9973

Protein	Log2FC	Average NPX	*P* Value	Adjusted *P* Value
**Serum: PHVDp1 vs PHVDp2**				
NEFL	1.0888	5.7718	0.0079	0.5265
RBKS	-1.0569	5.6700	0.0114	0.5265
PMVK	-0.9262	4.6089	0.0320	0.9814
ISLR2	-0.7634	4.3091	0.0512	0.9986
IL32	-0.6451	4.2740	0.0568	0.9986
SFRP1	0.5689	6.2684	0.1396	0.9986
VSTM1	0.5198	1.8421	0.1527	0.9986
SMOC1	0.3967	4.8257	0.1635	0.9986
FUT8	-0.5156	2.6395	0.1819	0.9986
CDH17	-0.3970	5.28921	0.1867	0.9986
**Serum: PHVDp1 vs PHVDp3**				
NEFL	1.4351	5.7718	0.0102	0.9456
VSTM1	0.8640	1.8421	0.0814	0.9945
DEFB4A	0.9126	4.4902	0.1023	0.9945
ISLR2	-0.7797	4.3091	0.1436	0.9945
SMOC1	0.5327	4.8257	0.1700	0.9945
IL32	-0.5747	4.2740	0.2128	0.9945
RBKS	-0.6796	5.6700	0.2315	0.9945
NXPH1	-0.3585	0.6700	0.2685	0.9945
SFRP1	0.5509	6.2684	0.2938	0.9945
TNFRSF13C	-0.3797	1.5303	0.3158	0.9945
**Serum: PHVDp2 vs PHVDp3**				
DEFB4A	1.2763	4.4902	0.0048	0.4467
PSG1	0.6295	2.3164	0.2580	0.9766
ABHD14B	-0.4839	3.0641	0.2862	0.9766
PLA2G10	0.4110	5.8624	0.3201	0.9766
PTS	0.4462	5.9466	0.3478	0.9766
TNFRSF13C	-0.2849	1.5303	0.3518	0.9766
KIF1BP	-0.4186	3.8851	0.3563	0.9766
PMVK	0.4251	4.6089	0.3707	0.9766
NPM1	-0.3265	6.7225	0.3741	0.9766
VSTM1	0.3441	1.8421	0.3900	0.9766
**Serum: IVH & IVHp vs 28 days of life**				
DSG3	1.3154	5.9457	0.0777	0.9846
PTS	1.6703	5.9466	0.0784	0.9846
PAEP	1.5804	2.4293	0.1170	0.9846
PSG1	1.6931	2.3164	0.1273	0.9846
ISLR2	1.2715	4.3091	0.1391	0.9846
NXPH1	0.6990	0.6700	0.1810	0.9846
TNFRSF13C	0.7407	1.5303	0.2250	0.9846
SNCG	0.8141	2.6413	0.2405	0.9846
PMVK	-0.8996	4.6089	0.3422	0.9846
CRIP2	0.8786	4.9117	0.3597	0.9846

Protein	Log2FC	Average NPX	*P* Value	Adjusted *P* Value
**Serum: IVH & IVHp vs 32 weeks GA**				
NPM1	-5.4943	6.7225	0.0010	0.0947
CRADD	-5.3371	5.4249	0.0053	0.2469
PMVK	-5.3398	4.6089	0.0133	0.4083
FKBP5	-4.3308	5.6880	0.0267	0.5261
PSME1	-3.3815	4.4051	0.0285	0.5261
TBCB	-5.1011	5.4333	0.0361	0.5549
PTPN1	-3.8271	5.8816	0.0450	0.5635
PLA2G10	-3.6276	5.8624	0.0530	0.5635
SRP14	-3.7538	4.7164	0.0551	0.5635
AKT1S1	-3.0389	2.5422	0.0752	0.6282
**Serum: IVH & IVHp vs term equivalent timepoint**				
NPM1	-3.4700	6.7225	0.0114	0.5636
PLA2G10	-3.8688	5.8624	0.0122	0.5636
SMOC1	-2.6084	4.8257	0.0259	0.6052
CRADD	-3.3379	5.4249	0.0338	0.6052
RBKS	-3.3838	5.6700	0.0481	0.6052
GPNMB	-2.3861	5.8473	0.0537	0.6052
PAEP	3.3470	2.4293	0.0756	0.6052
FGFR2	-1.8623	5.0611	0.0835	0.6052
DEFB4A	-2.9055	4.4902	0.0841	0.6052
CD63	-2.3617	6.9384	0.0845	0.6052
**Serum: 28 days of life vs 32 weeks GA**				
NPM1	-5.3708	6.7225	0.0004	0.0386
CRADD	-4.6166	5.4249	0.0080	0.3070
PMVK	-4.4402	4.6089	0.0234	0.3070
PSME1	-3.0637	4.4051	0.0290	0.3070
SRP14	-3.8782	4.7164	0.0293	0.3070
PTPN1	-3.6880	5.8816	0.0336	0.3070
RBKS	-4.0109	5.6700	0.0341	0.3070
FKBP5	-3.7183	5.6880	0.0362	0.3070
CDH17	-2.8555	5.2892	0.0365	0.3070
GPNMB	-2.8145	5.8473	0.0395	0.3070
**Serum: 28 days of life vs term equivalent timepoint**				
SMOC1	-3.0203	4.8257	0.0032	0.1904
NPM1	-3.3465	6.7225	0.0054	0.1904
PLA2G10	-3.7031	5.8624	0.0062	0.1904
RBKS	-3.9397	5.6700	0.0087	0.2005
GPNMB	-2.7074	5.8473	0.0125	0.2313
FGFR2	-2.2732	5.0611	0.0160	0.2400
CD63	-2.7913	6.9384	0.0200	0.2400
CDH17	-2.4964	5.2892	0.0211	0.2400
ADGRB3	-2.3176	5.4937	0.0234	0.2400
CDH15	-3.0821	8.3480	0.0408	0.3754

**Protein**	**Log2FC**	**Average NPX**	***P* Value**	**Adjusted *P* Value**
**Serum: 32 weeks GA vs term equivalent timepoint**				
FKBP5	3.1308	5.6880	0.1439	0.9918
TDGF1	3.5374	4.5991	0.2456	0.9918
PMVK	2.6999	4.6089	0.2529	0.9918
NPM1	2.0243	6.7225	0.2679	0.9918
EREG	2.8198	2.9359	0.2701	0.9918
HMOX2	1.8137	4.0660	0.2825	0.9918
NAA10	2.3789	4.2379	0.2883	0.9918
TBCB	2.7778	5.4333	0.2978	0.9918
SMOC1	-1.4967	4.8257	0.3371	0.9918
CRADD	1.9992	5.4249	0.3402	0.9918

Table S7: Top 10 differentially expressed proteins between surviving and deceased patients at defined timepoints based on the urine data. Group comparison using the *limma* R package [3] to identify significant changes in the NPX values.

Protein	Log2FC	Average NPX	*P* Value	Adjusted *P* Value
**Timepoint IVH**				
HSP90B1	0.3845	-0.6117	0.0485	0.9652
LTBP3	-0.6190	2.7819	0.0523	0.9652
ATP6V1F	0.3396	-0.3033	0.0665	0.9652
EIF4B	-0.6025	0.3105	0.1049	0.9652
CRADD	-0.4613	0.8348	0.1123	0.9652
FUT8	0.2754	-1.2293	0.1358	0.9652
NXPH1	-0.2909	0.3174	0.1370	0.9652
KIRREL2	-0.3327	1.2216	0.1442	0.9652
TBCB	-0.3210	-0.3389	0.1856	0.9652
ISLR2	-0.3717	1.6447	0.2066	0.9652
**Timepoint IVHp**				
IL15	-0.7486	0.5405	0.0006	0.0364
FKBP5	-1.2300	4.4774	0.0007	0.0364
PFDN2	-0.3550	0.1961	0.0016	0.0518
PTPN1	-0.8296	2.6298	0.0033	0.0778
ANXA10	-1.0416	1.4432	0.0049	0.0857
HMOX2	-0.5720	2.1718	0.0063	0.0857
EIF4B	-0.7632	-0.3986	0.0071	0.0857
NAA10	-0.5435	0.8265	0.0076	0.0857
SNCG	-0.8158	2.5116	0.0083	0.0857
FHIT	-0.5249	-1.6790	0.0119	0.1101
**Timepoint PHVD**				
FGFR2	1.0005	2.6138	2.1883 e^{-5}	0.0012
LTBP3	1.7667	3.5041	2.7465 e^{-5}	0.0012
TNFRSF13C	0.8992	1.5865	4.4555 e^{-5}	0.0013
FKBP5	-1.8565	3.8739	9.2685 e^{-5}	0.0017
CDH15	1.7411	2.9752	9.6396 e^{-5}	0.0017
SFRP1	1.5274	3.7714	0.0014	0.0221
ADAM15	0.8495	2.9638	0.0017	0.0233
NEFL	-1.2951	2.1933	0.0054	0.0627
CD33	0.4541	-1.5101	0.0101	0.0890
BST2	0.5532	1.2038	0.0106	0.0890
**Timepoint PHVDp1**				
FKBP5	-2.6017	3.1671	5.0097 e^{-8}	4.6089 e^{-6}
PTPN1	-1.3590	2.1435	0.0001	0.0048
HMOX2	-1.0503	1.8897	0.0004	0.0132
ILKAP	-0.9816	0.7315	0.0005	0.0132
TNFRSF13C	0.9879	2.0387	0.0008	0.0156
LTBP3	1.4529	4.0161	0.0011	0.0182
MAD1L1	-0.9227	1.0166	0.0037	0.0486
NEFL	-1.2120	1.7368	0.0095	0.1097
HSP90B1	-0.3715	-0.7079	0.0132	0.1317
ADAM15	0.8117	3.2785	0.0143	0.1317

**Protein**	**Log2FC**	**Average NPX**	***P* Value**	**Adjusted *P* Value**
**Timepoint PHVDp2**				
IL32	-1.3517	0.7562	4.1326 e^{-6}	0.0003
NEFL	-1.6628	1.2508	4.0742 e^{-5}	0.0018
FKBP5	-1.9618	2.3681	0.0003	0.0104
PTPN1	-1.2816	1.9961	0.0021	0.0499
AKT1S1	-0.5763	-0.5999	0.0039	0.0718
ILKAP	-0.9319	0.6759	0.0071	0.0868
PAEP	-0.3483	-0.6789	0.0073	0.0868
CRADD	-0.5642	-0.1147	0.0075	0.0868
HMOX2	-0.8658	1.9311	0.0120	0.1126
MAD1L1	-0.9623	1.1239	0.0122	0.1126
**Timepoint PHVDp3**				
FKBP5	-2.0973	1.5064	2.2568 e^{-6}	0.0002
CD302	-2.7142	2.8576	9.3157 e^{-6}	0.0004
SMOC1	-0.6936	-0.1859	0.0001	0.0048
FKBP7	-0.6065	-0.6659	0.0011	0.0232
PTPN1	-1.2910	1.3269	0.0012	0.0232
PAEP	-0.3659	-0.7336	0.0029	0.0384
NEFL	-1.0149	0.5114	0.0031	0.0384
ASGR1	-0.9814	2.0910	0.0033	0.0384
SNCG	-1.3058	1.9640	0.0043	0.0439
CLSTN1	-0.3953	-1.2160	0.0061	0.0545
**Timepoint IVH & IVHp**				
FKBP5	-0.9769	4.4226	0.0002	0.0090
IL15	-0.5984	0.6695	0.0002	0.0090
EIF4B	-0.8377	-0.1454	0.0002	0.0090
PTPN1	-0.7479	2.8313	0.0005	0.0124
CRADD	-0.5579	0.3619	0.0009	0.0174
SNCG	-0.7570	2.5951	0.0015	0.0244
ANXA10	-0.8141	1.6012	0.0021	0.0285
NXPH1	-0.3123	0.0501	0.0037	0.0436
PFDN2	-0.2735	0.2708	0.0053	0.0488
HMOX2	-0.4517	2.2830	0.0056	0.0488
**Timepoint 28 days of life**				
IL32	-1.5376	0.5853	2.9739 e^{-7}	2.736 e^{-5}
KLB	-0.6431	-0.2321	0.0040	0.1865
FKBP5	-1.7702	2.6401	0.0080	0.2463
PTPN1	-1.1589	2.0044	0.0177	0.3322
ILKAP	-0.8498	0.6510	0.0180	0.3322
HMOX2	-0.9134	1.8454	0.0313	0.4277
ASGR1	-0.8554	2.5120	0.0325	0.4277
CARHSP1	-0.5304	-0.5192	0.0389	0.4480
PAEP	-0.3474	-0.6495	0.0666	0.6170
NEFL	-1.1909	1.6678	0.0670	0.6170

**Protein**	**Log2FC**	**Average NPX**	***P* Value**	**Adjusted *P* Value**
**Timepoint 32 weeks GA**				
IL32	-1.5712	0.7070	$7.5384\ e^{-5}$	0.0069
FKBP5	-2.0951	2.4011	0.0003	0.0165
PTPN1	-1.3887	2.1047	0.0039	0.1214
ANXA10	-1.5234	1.5745	0.0069	0.1594
HMOX2	-1.0023	1.9749	0.0091	0.1681
AKT1S1	-0.5818	-0.7129	0.0130	0.1998
IL15	-0.6293	0.1389	0.0219	0.2648
NAA10	-0.9072	0.7187	0.0230	0.2648
CARHSP1	-0.8088	-0.3926	0.0259	0.2648
IFI30	-0.9588	4.6622	0.0291	0.2685

Table S8: Top 10 differentially expressed proteins between surviving and deceased patients at defined timepoints based on the serum data. Group comparison using the *limma* R package [3] to identify significant changes in the NPX values.

Protein	Log2FC	Average NPX	*P* Value	Adjusted *P* Value
**Timepoint IVH**				
PRTFDC1	-1.4841	1.0402	3.4316 e^{-7}	3.1570 e^{-5}
DPEP2	0.7231	1.3300	0.0001	0.0035
FKBP5	-1.0297	6.0310	0.0001	0.0035
SNCG	-1.1418	3.0927	0.0001	0.0035
AKT1S1	-0.8833	2.6540	0.0005	0.0093
PMVK	-1.2194	3.9717	0.0029	0.0445
PFDN2	-0.4965	2.0030	0.0035	0.0445
RPS6KB1	-0.7284	0.1431	0.0038	0.0445
DSG3	0.7599	6.1827	0.0080	0.0826
PTPN1	-0.5879	5.9768	0.0115	0.0982
**Timepoint IVHp**				
IL15	-0.8778	3.3573	3.4414 e^{-6}	0.0003
DPEP2	0.7366	1.4094	8.5908 e^{-5}	0.0039
FKBP5	-0.9604	6.3009	0.0003	0.0111
RBKS	-1.2120	5.7290	0.0017	0.0410
ASGR1	-0.4498	3.8225	0.0061	0.1094
IFNL1	0.6603	0.8129	0.0071	0.1094
SNCG	-0.6831	2.8779	0.0088	0.1165
PTS	-0.6563	6.3527	0.0206	0.1972
ABHD14B	0.6833	3.3341	0.0226	0.1972
ANXA10	-0.7230	2.7743	0.0264	0.1972
**Timepoint PHVD**				
GPNMB	1.0092	5.9319	2.1779 e^{-12}	2.0037 e^{-10}
DSG3	1.6208	6.1486	1.7435 e^{-11}	8.0202 e^{-10}
FGFR2	1.1118	5.1424	1.2934 e^{-10}	3.9664 e^{-9}
CDH15	1.6626	8.2891	4.3027 e^{-9}	8.8233 e^{-8}
NEFL	-1.8891	6.7268	4.7953 e^{-9}	8.8233 e^{-8}
IL15	-0.9193	2.7746	1.5154 e^{-6}	2.3236 e^{-5}
ADAM15	0.9912	8.4597	2.7770 e^{-6}	3.5665 e^{-5}
IL32	0.8068	3.8891	3.1013 e^{-6}	3.5665 e^{-5}
KIRREL2	0.8175	4.4091	6.0091 e^{-5}	0.0006
CDH17	0.6986	5.8341	0.0001	0.0010
**Timepoint PHVDp1**				
FGFR2	0.9714	5.2138	4.0317 e^{-10}	3.7091 e^{-8}
CDH15	1.5302	8.4184	4.8292 e^{-9}	2.2214 e^{-7}
GPNMB	0.8109	5.9911	2.0790 e^{-8}	6.3758 e^{-7}
ADAM15	1.0712	8.5233	7.5060 e^{-8}	1.7263 e^{-6}
IL15	-0.9633	2.4579	1.1133 e^{-7}	2.0485 e^{-6}
PLA2G10	1.1467	5.9414	8.7863 e^{-7}	1.3472 e^{-5}
DSG3	1.2339	6.2983	1.0436 e^{-6}	1.3717 e^{-5}
BST2	0.7897	4.1693	1.9018 e^{-6}	1.9505 e^{-5}
NEFL	-1.4698	6.5823	1.9080 e^{-6}	1.9505 e^{-5}
TPPP3	0.8962	0.4227	0.0001	0.0016

**Protein**	**Log2FC**	**Average NPX**	***P* Value**	**Adjusted *P* Value**
**Timepoint PHVDp2**				
PAEP	-1.7592	1.5515	3.1708 e^{-6}	0.0002
CRIP2	1.4551	5.2804	1.9291 e^{-5}	0.0008
IFNL1	0.9330	1.0791	0.0001	0.0054
ABHD14B	-1.5846	2.8484	0.0004	0.0103
DPEP2	-0.9280	1.8451	0.0022	0.0402
TNFRSF13C	0.6213	1.3567	0.0033	0.0402
CD302	0.6857	5.4543	0.0033	0.0402
PSG1	-1.1513	1.0542	0.0035	0.0402
NDRG1	0.4972	0.6184	0.0072	0.0744
GSTP1	-0.4629	0.1730	0.0106	0.0980
**Timepoint PHVDp3**				
IFNL1	1.0703	1.2907	3.4881 e^{-7}	3.2090 e^{-5}
ASGR1	-0.5216	3.4063	3.2245 e^{-5}	0.0014
SNCG	-0.8120	2.1267	0.0001	0.0055
PLA2G10	0.9057	6.7073	0.0002	0.0055
PPP3R1	-0.6553	3.2775	0.0005	0.0092
FKBP7	-0.4066	0.3078	0.0017	0.0264
PSG1	-1.0681	0.6923	0.0024	0.0319
ADAM15	0.5510	8.9272	0.0042	0.0492
IL32	0.4179	5.0625	0.0050	0.0517
PTPN1	-0.9084	5.8283	0.0074	0.0687
**Timepoint IVH & IVHp**				
DPEP2	0.7255	1.3793	1.3193 e^{-7}	1.2138 e^{-5}
FKBP5	-0.9715	6.1784	9.8651 e^{-7}	4.5379 e^{-5}
SNCG	-0.8422	2.9706	3.3497 e^{-5}	0.0010
IL15	-0.5662	3.4307	8.1851 e^{-5}	0.0018
PRTFDC1	-0.8022	1.1362	0.0003	0.0071
RBKS	-0.9607	5.8668	0.0011	0.0175
PPP3R1	-0.3923	4.0328	0.0021	0.0287
IFNL1	0.5431	0.7747	0.0028	0.0331
CRIP2	-0.5560	4.7373	0.0043	0.0428
AKT1S1	-0.5900	2.6644	0.0046	0.0428
**Timepoint 28 days of life**				
CRIP2	1.5993	5.0422	0.0001	0.0117
PLA2G10	1.2684	6.3909	0.0002	0.0117
PAEP	-1.4493	1.8675	0.0074	0.1775
ADGRB3	0.5229	5.5954	0.0077	0.1775
PSG1	-1.1998	1.1063	0.0110	0.2035
TNFRSF13C	0.5151	1.0319	0.0214	0.3281
ISLR2	0.8386	4.0887	0.0300	0.3955
IFNL1	0.6854	0.9323	0.0447	0.4125
KIRREL2	0.5123	4.5742	0.0452	0.4125
VSTM1	0.7234	1.6203	0.0468	0.4125

Protein	Log2FC	Average NPX	*P* Value	Adjusted *P* Value
**Timepoint 32 weeks GA**				
CEACAM3	-0.7579	-0.0159	0.0004	0.0291
HMOX2	-1.2891	3.8333	0.0006	0.0291
FHIT	-1.5808	0.9319	0.0028	0.0572
PFDN2	-0.8935	1.3623	0.0029	0.0572
GSTP1	-0.8702	0.1659	0.0043	0.0572
DPEP2	-1.2598	1.9101	0.0046	0.0572
IFNL1	0.9969	0.9910	0.0048	0.0572
ECE1	-1.1879	0.9887	0.0054	0.0572
PSME1	-1.2613	4.2596	0.0061	0.0572
ABHD14B	-1.7377	2.8076	0.0070	0.0572
**Timepoint term equivalent age**				
IFNL1	1.3310	1.4403	0.0005	0.0360
PRTFDC1	-2.0328	0.9178	0.0011	0.0360
FHIT	-2.0899	0.9752	0.0011	0.0360
FCAR	-1.1309	1.3813	0.0021	0.0402
CARHSP1	-1.7973	0.8095	0.0021	0.0402
PSME1	-1.6622	4.5200	0.0031	0.0449
PMVK	-2.5737	4.4820	0.0034	0.0449
PFDN2	-0.8948	1.3613	0.0044	0.0508
CRADD	-2.0665	5.5074	0.0056	0.0572
PPP3R1	-0.9049	3.2637	0.0073	0.0678

References S1

[1] W. E. Johnson, C. Li, and A. Rabinovic, "Adjusting batch effects in microarray expression data using empirical Bayes methods," *Biostatistics*, vol. 8, no. 1, pp. 118–127, Jan. 2007, doi: 10.1093/biostatistics/kxj037.

[2] J. T. Leek and J. D. Storey, "Capturing Heterogeneity in Gene Expression Studies by Surrogate Variable Analysis," *PLoS Genet*, vol. 3, no. 9, p. e161, Sep. 2007, doi: 10.1371/journal.pgen.0030161.

[3] M. E. Ritchie *et al.*, "limma powers differential expression analyses for RNA-sequencing and microarray studies," *Nucleic Acids Res*, vol. 43, no. 7, pp. e47–e47, Apr. 2015, doi: 10.1093/nar/gkv007.

[4] R. C. Gentleman *et al.*, "Bioconductor: open software development for computational biology and bioinformatics," *Genome Biol*, vol. 5, no. 10, p. R80, 2004, doi: 10.1186/gb-2004-5-10-r80.

[5] R Core Team, "R: A language and environment for statistical computing," 2022, *R Foundation for Statistical Computing, Vienna, Austria*.

[6] D. Ruiz-Perez, H. Guan, P. Madhivanan, K. Mathee, and G. Narasimhan, "So you think you can PLS-DA?," *BMC Bioinformatics*, vol. 21, no. S1, p. 2, Dec. 2020, doi: 10.1186/s12859-019-3310-7.

[7] J. Jansson *et al.*, "Metabolomics Reveals Metabolic Biomarkers of Crohn's Disease," *PLoS One*, vol. 4, no. 7, p. e6386, Jul. 2009, doi: 10.1371/journal.pone.0006386.

[8] I.-G. Chong and C.-H. Jun, "Performance of some variable selection methods when multicollinearity is present," *Chemometrics and Intelligent Laboratory Systems*, vol. 78, no. 1–2, pp. 103–112, Jul. 2005, doi: 10.1016/j.chemolab.2004.12.011.

[9] L. E. Broughton-Neiswanger, S. M. Rivera-Velez, M. A. Suarez, J. E. Slovak, J. K. Hwang, and N. F. Villarino, "Pharmacometabolomics with a combination of PLS-DA and random forest algorithm analyses reveal meloxicam alters feline plasma metabolite profiles," *J Vet Pharmacol Ther*, vol. 43, no. 6, pp. 591–601, Nov. 2020, doi: 10.1111/jvp.12884.

[10] T. Chen *et al.*, "Random Forest in Clinical Metabolomics for Phenotypic Discrimination and Biomarker Selection," *Evidence-Based Complementary and Alternative Medicine*, vol. 2013, pp. 1–11, 2013, doi: 10.1155/2013/298183.

[11] S. M. Rivera-Velez *et al.*, "Repeated administration of the NSAID meloxicam alters the plasma and urine lipidome," *Sci Rep*, vol. 9, no. 1, p. 4303, Mar. 2019, doi: 10.1038/s41598-019-40686-4.

[12] L. E. Broughton-Neiswanger, S. M. Rivera-Velez, M. A. Suarez, J. E. Slovak, J. K. Hwang, and N. F. Villarino, "Pharmacometabolomics with a combination of PLS-DA and random forest algorithm analyses reveal meloxicam alters feline plasma metabolite profiles," *J Vet Pharmacol Ther*, vol. 43, no. 6, pp. 591–601, Nov. 2020, doi: 10.1111/jvp.12884.

[13] C. M. Andersen and R. Bro, "Variable selection in regression—a tutorial," *J Chemom*, vol. 24, no. 11–12, pp. 728–737, Nov. 2010, doi: 10.1002/cem.1360.

[14] J. Friedman, T. Hastie, and R. Tibshirani, "Regularization Paths for Generalized Linear Models via Coordinate Descent," *J Stat Softw*, vol. 33, no. 1, 2010, doi: 10.18637/jss.v033.i01.

[15] G. D. Garson, "Interpreting neural-network connection weights," *AI Expert*, vol. 6, no. 4, pp. 46–51, Apr. 1991.

[16] M. Kuhn, “Building Predictive Models in *R* Using the **caret** Package,” *J Stat Softw*, vol. 28, no. 5, 2008, doi: 10.18637/jss.v028.i05.

[17] M. Kuhn and K. Johnson, *Applied Predictive Modeling*. New York, NY: Springer New York, 2013. doi: 10.1007/978-1-4614-6849-3.

[18] D. Ruiz-Perez, H. Guan, P. Madhivanan, K. Mathee, and G. Narasimhan, “So you think you can PLS-DA?,” *BMC Bioinformatics*, vol. 21, no. S1, p. 2, Dec. 2020, doi: 10.1186/s12859-019-3310-7.

[19] L. Fagerberg *et al.*, “Analysis of the Human Tissue-specific Expression by Genome-wide Integration of Transcriptomics and Antibody-based Proteomics,” *Molecular & Cellular Proteomics*, vol. 13, no. 2, pp. 397–406, Feb. 2014, doi: 10.1074/mcp.M113.035600.

[20] Y. Lai *et al.*, “Identification of immune microenvironment subtypes and signature genes for Alzheimer’s disease diagnosis and risk prediction based on explainable machine learning,” *Front Immunol*, vol. 13, Dec. 2022, doi: 10.3389/fimmu.2022.1046410.

[21] Y. Huang, Z. Zhang, and L. Chen, “Diagnosis and prognosis of serum Fut8 for epilepsy and refractory epilepsy in children,” *PLoS One*, vol. 18, no. 4, p. e0284239, Apr. 2023, doi: 10.1371/journal.pone.0284239.
